# Rational Design of Putative Dual-Target Opioid-Dopaminergic Casomorphin–Ranatensin Hybrid Peptides: Computational Modeling and Preliminary Antiproliferative Evaluation

**DOI:** 10.3390/molecules31183221

**Published:** 2026-09-12

**Authors:** Krystian Małek, Adrian Górski, Łukasz Szeleszczuk, Anna K. Laskowska, Zuzanna Markowska, Sebastian Granica, Anna Pogorzelska, Piotr Koch, Natalia Pielaszkiewicz, Jagoda Michniewicz, Wojciech Kamysz, Małgorzata Milczarek, Karol Sikora, Patrycja Kleczkowska

**Affiliations:** 1 Department of Biomedical Research, National Medicines Institute, 00-725 Warsaw, Poland; krystianmalekkr@gmail.com (K.M.); a.pogorzelska@nil.gov.pl (A.P.); m.milczarek@nil.gov.pl (M.M.); 2 Department of Inorganic Chemistry, Faculty of Pharmacy, Medical University of Gdansk, 80-416 Gdansk, Poland; adrian.gorski@gumed.edu.pl (A.G.); kamysz@gumed.edu.pl (W.K.); karol.sikora@gumed.edu.pl (K.S.); 3 Department of Organic and Physical Chemistry, Medical University of Warsaw, 02-097 Warsaw, Poland; lukasz.szeleszczuk@wum.edu.pl; 4 Department of Pharmaceutical Microbiology, Medical University of Warsaw, 02-091 Warsaw, Poland; anna.laskowska@wum.edu.pl; 5 Department of Pharmaceutical Biology, Medical University of Warsaw, 02-097 Warsaw, Poland; zuzanna.czarnomska@wum.edu.pl (Z.M.); sebastian.granica@wum.edu.pl (S.G.); 6 Maria Sklodowska-Curie Medical Academy in Warsaw, 03-411 Warsaw, Poland; piotrkoch@icloud.com (P.K.); npielaszkiewicz@gmail.com (N.P.); jagoda.michniewicz@uczelniamedyczna.com.pl (J.M.); 7 Department of Nursing and Other Health Professions, Center of Postgraduate Medical Education, 01-826 Warsaw, Poland

**Keywords:** casomorphins, hybrid peptides, antiproliferative activity, molecular docking, molecular dynamics

## Abstract

Despite advances in oncology, cancer remains a leading cause of mortality worldwide, and both μ-opioid receptor (MOR) and dopamine D2 receptor (D2R) signaling have been implicated in the regulation of tumor proliferation, survival, and progression, positioning them as attractive targets for multifunctional anticancer strategies. In the present study, a series of novel casomorphin–ranatensin hybrid peptides were designed, synthesized, and evaluated as putative dual-target anticancer agents combining MOR and D2R pharmacophores. Among the evaluated analogues, KAZO_5.2 and KAZO_7.2 reduced viability of HCT116 colorectal cancer cells in a dose- and time-dependent manner, with a modest selectivity ratio compared to non-tumorigenic MCF 10A cells. Both compounds showed favorable docking scores toward MOR and D2R, while single 100 ns molecular dynamics trajectories showed that the peptides remained associated with their respective receptor models over the simulated timescale. Partial reversal of the viability-reducing effect by naloxone suggested involvement of opioid-sensitive pathways, while only low baseline MOR expression was detected in HCT116 cells under our experimental conditions, consistent with a possible contribution of additional receptor-mediated mechanisms to the observed activity. Neither compound induced pronounced apoptosis or cell cycle arrest, suggesting cytostatic rather than cytotoxic effects. Both peptides exhibited negligible hemolytic activity and high proteolytic stability in human plasma, with intact peptides remaining predominant after 24 h of incubation at 37 °C. These findings identify casomorphin–ranatensin hybrids as a promising scaffold for dual-target anticancer peptide development, warranting further structural optimization and mechanistic investigation.

## 1. Introduction

Despite decades of intensive research and substantial advances in diagnostics and therapy, cancer remains one of the leading causes of morbidity and mortality worldwide [1,2]. The clinical management of many malignancies is still dominated by fundamental challenges, including late-stage diagnosis, tumor heterogeneity, rapid disease progression, and the emergence of intrinsic or acquired resistance to therapy. As a result, for a significant proportion of patients, current treatment strategies provide only limited and often transient clinical benefit, underscoring the persistent gap between mechanistic understanding of cancer biology and durable therapeutic success [3,4,5]. In this sense, anticancer drug development continues to resemble a struggle against a constantly adapting adversary rather than a linear path toward definitive solutions.

These limitations highlight the inadequacy of single-target approaches to address the biological complexity of cancer and emphasize the need for therapeutic strategies capable of interfering with multiple, interconnected signaling pathways. In this context, multifunctional peptide hybrids have emerged as a promising class of compounds [6,7,8,9], offering the possibility to integrate distinct pharmacophores within a single molecular framework and thereby achieve synergistic biological effects.

Among multifunctional scaffolds, β-casomorphins, including β-casomorphin-5 (BCM-5) and β-casomorphin-7 (BCM-7), stand out due to their structural versatility, opioid activity, and potential to modulate cancer-related processes. In this context, accumulating evidence indicates that these peptides exert broader biological effects, including modulation of cell proliferation, apoptosis, and immune responses [10,11,12,13,14,15,16]. Their favorable structural properties, such as conformational flexibility and compatibility with peptide engineering, make them attractive scaffolds for the design of novel hybrid molecules with potential antiproliferative activity. Notably, β-casomorphin-7 has recently been shown to suppress colorectal cancer development and metastasis by enhancing anti-tumoral immunity in vivo, further supporting the relevance of casomorphin-derived scaffolds in oncology [17].

In parallel, dopamine D2 receptors (D2R) have gained attention beyond their established role in neuropsychiatric disorders and are now recognized as important regulators of tumor-associated processes, including angiogenesis, cell migration, and cancer cell survival [18,19,20,21]. Pharmacological modulation of D2R has been shown in multiple experimental models to suppress tumor growth and to enhance the efficacy of other anticancer interventions, positioning this receptor as an attractive target in combination-based therapeutic approaches. In this context, the ranatensin-derived GHFM fragment was selected as the dopaminergic pharmacophore based on evidence demonstrating the ability of ranatensin to modulate D2R-expressing pancreatic cancer cells [22] and on the precedent established by LENART01, a structurally related opioid-dopaminergic chimera incorporating the same fragment, which demonstrated binding activity at both opioid and dopamine D2 receptors [23].

**Figure 1:**
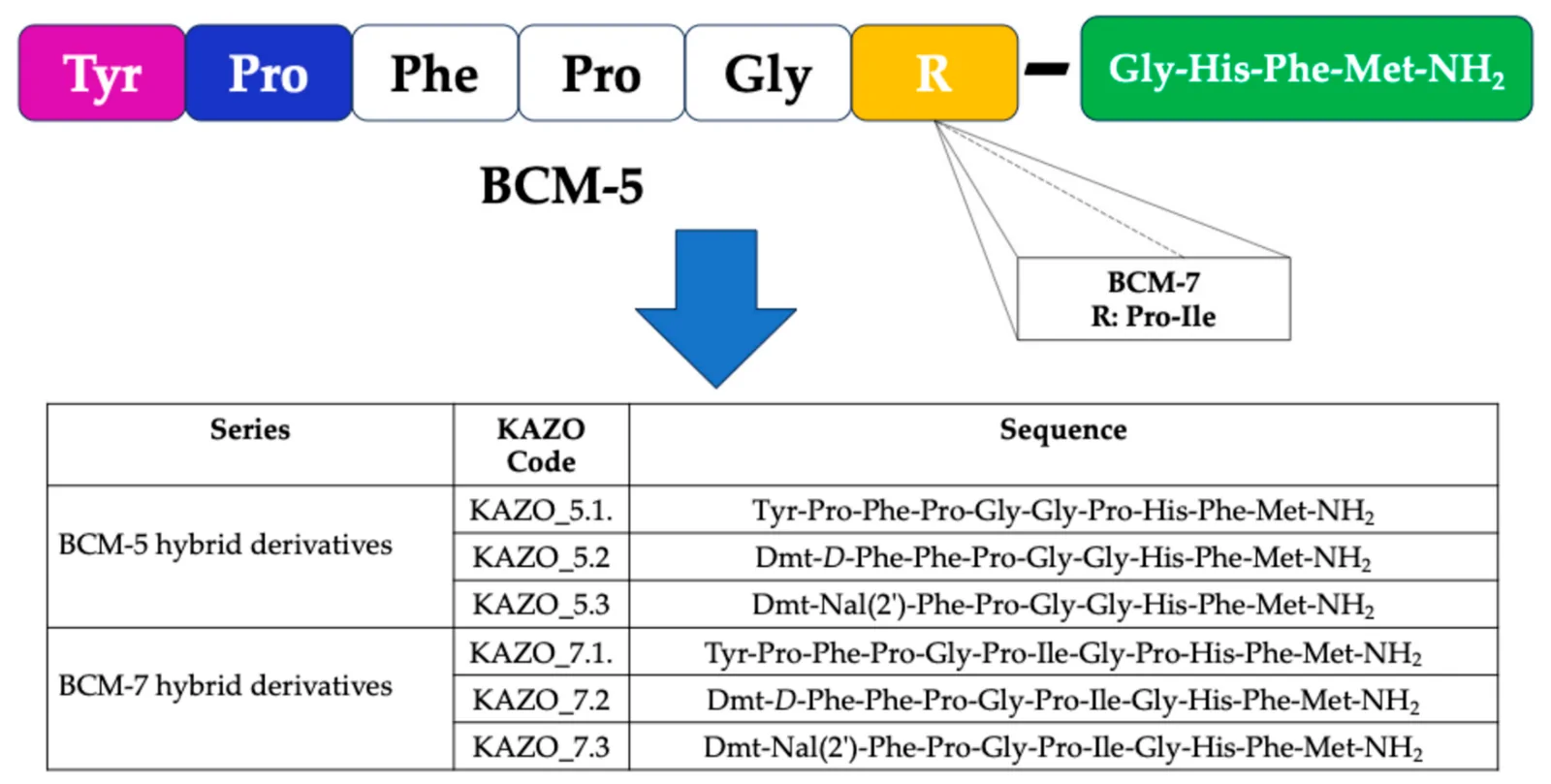
Chemical composition of hybrid peptides derived from BCM-5 (KAZO_5; YPFPG) and BCM-7 (KAZO_7; YPFPGPI), targeting opioid receptors, with modifications at the first two N-terminal residues and conjugated with the GHFM (Gly-His-Phe-Met; green) motif derived from the modified ranatensin fragment, targeting D2R (as used in LENART01 [23,24]).

In the present study, a new series of peptide hybrids was designed and synthesized, incorporating BCM-5 or BCM-7 as one pharmacophoric component, while the C-terminal fragment was selected to theoretically enable interaction with the dopaminergic system (Figure 1). This design strategy aims to generate molecules with a dual biological profile, combining opioid-like activity with modulation of dopaminergic signaling, which may translate into enhanced anticancer effects through complementary mechanisms of action. Therefore, the aim of the study was to evaluate the antiproliferative activity of the newly synthesized casomorphin-based hybrid peptides.

## 2. Results and Discussion

### 2.1. Molecular Docking to MOR and D2R

Molecular docking studies revealed substantial differences in the predicted binding modes and relative docking performance of the investigated KAZO peptide analogues toward the murine μ-opioid receptor (MOR, PDB ID: 5C1M) and human dopamine D2 receptor (D2R, PDB ID: 7JVR) (Table 1). All MOR residue numbers and residue-level structural interpretations discussed below therefore refer specifically to the murine 5C1M structure.

To validate the docking setup, the co-crystallized ligands used to define the receptor grids were redocked into their respective binding sites. Redocking of BU72 into MOR (PDB ID: 5C1M) reproduced the crystallographic pose with a heavy-atom RMSD of 0.410 Å, whereas redocking of bromocriptine into D2R (PDB ID: 7JVR) yielded a heavy-atom RMSD of 0.915 Å. The close agreement between the crystallographic and redocked poses supports the suitability of the binding-site definition and docking setup used for the subsequent docking calculations (Figure S1).

**Figure 2:**
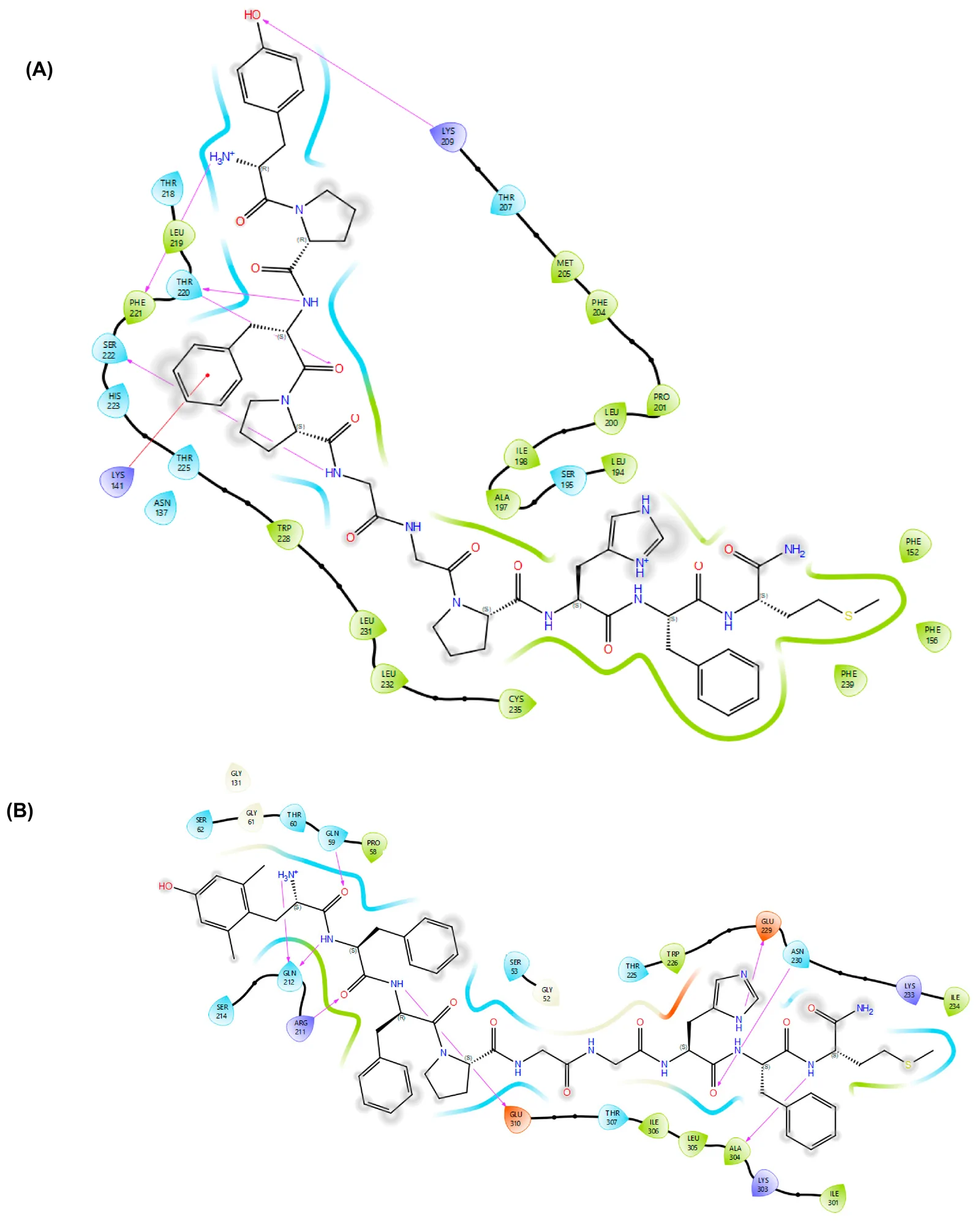

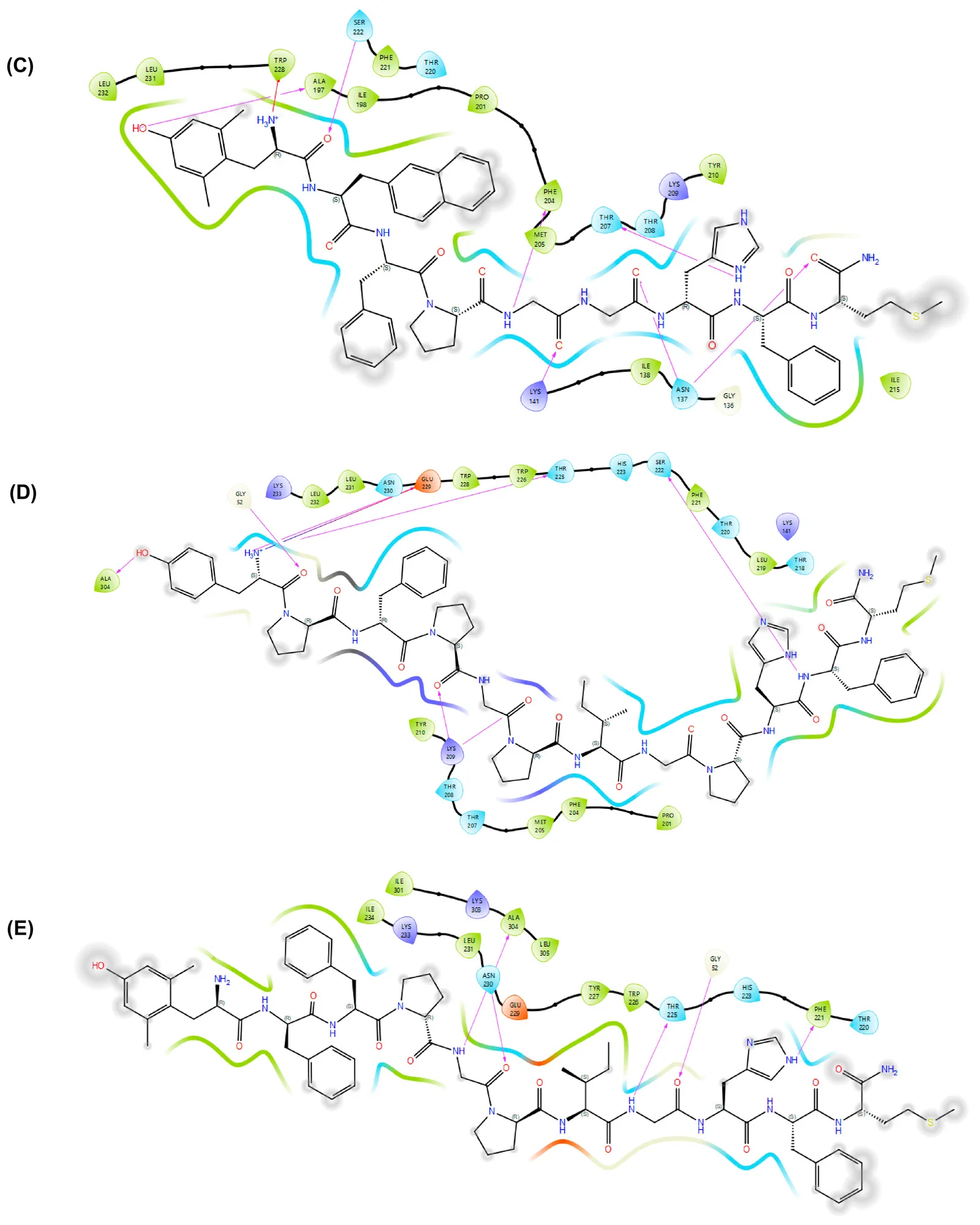

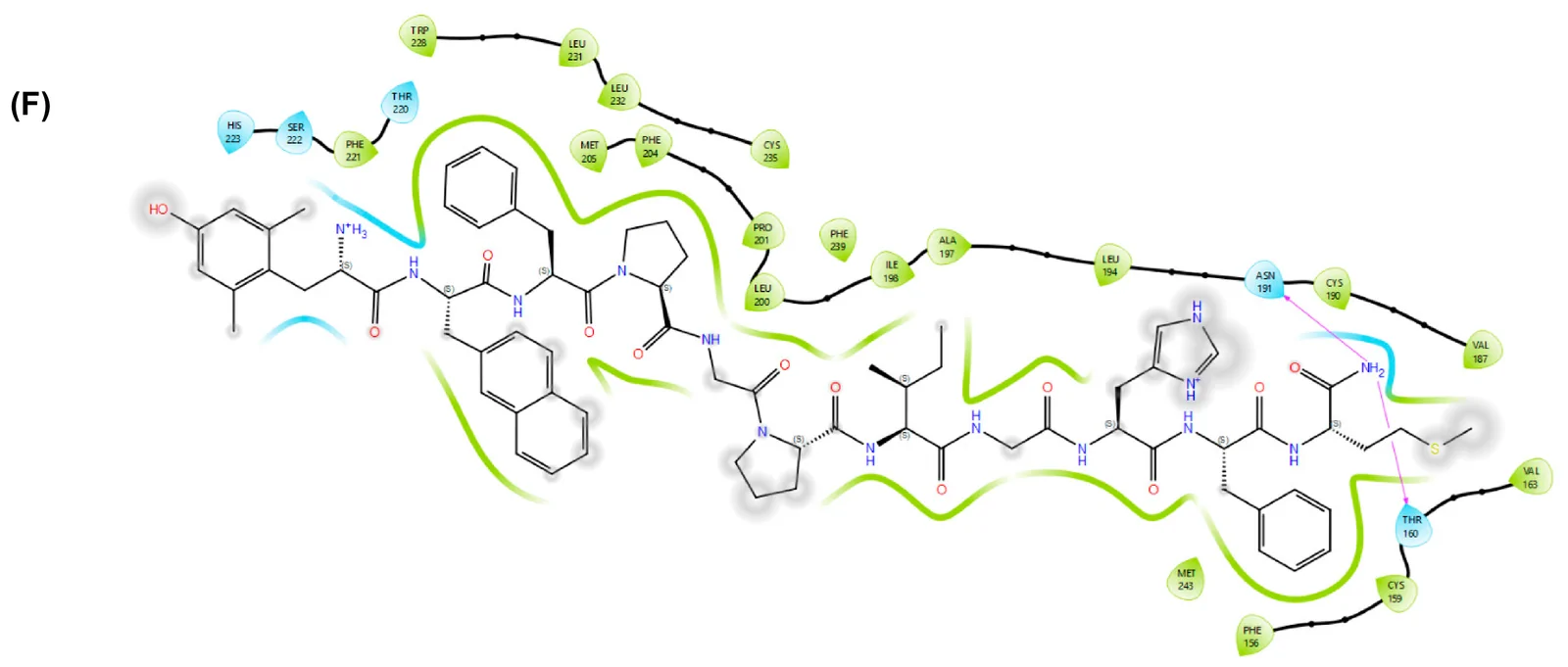
Two-dimensional ligand–receptor interaction diagrams for the investigated peptides in complex with the murine μ-opioid receptor (MOR, PDB ID: 5C1M) obtained using Glide SP-Peptide docking. Hydrogen bonds are represented by purple arrows, while π-type interactions are shown as red arrows. Hydrophobic interactions and surrounding receptor residues contributing to ligand stabilization within the binding pocket are also indicated. (**A**) KAZO_5.1; (**B**) KAZO_5.2; (**C**) KAZO_5.3; (**D**) KAZO_7.1; (**E**) KAZO_7.2; (**F**) KAZO_7.3.

In the case of MOR, among all investigated ligands, KAZO_5.2 and KAZO_7.2 exhibited the most favorable docking scores. In contrast, KAZO_7.1 showed a less favorable docking score despite retaining several stabilizing receptor interactions, suggesting that ligand geometry and conformational organization play critical roles in MOR recognition (Table 1, Figure 2).

KAZO_5.1 adopted a relatively extended binding conformation within the MOR binding pocket. Although the ligand formed multiple hydrogen-bond interactions involving residues THR220, PHE221, and SER222, as well as aromatic interactions in the vicinity of LYS141, the peptide appeared only partially embedded within the receptor cavity. The C-terminal region remained relatively exposed to the solvent-accessible receptor surface (Figure 2A). This likely resulted in increased conformational flexibility and reduced complex stability. A substantial improvement in docking performance was observed after the introduction of Dmt and D-Phe residues in KAZO_5.2. Indeed, KAZO_5.2 demonstrated one of the most favorable docking scores and formed an extensive hydrogen-bond network within the MOR binding pocket (Table 1, Figure 2B). The ligand formed an extensive hydrogen-bond network involving GLN59, ARG211, GLN212, GLU229, GLU310, and ALA304. In contrast to KAZO_5.1, the N-terminal pharmacophoric region of KAZO_5.2 adopted a significantly more compact and deeply embedded orientation within the receptor binding pocket (Figure 2B). The presence of Dmt likely enhanced hydrophobic complementarity and aromatic packing interactions, while D-Phe promoted a more conformationally organized ligand geometry. Importantly, both the central and C-terminal fragments of the peptide actively participated in receptor recognition, leading to improved stabilization of the entire ligand within the MOR binding cavity. Replacement of D-Phe with the bulkier Nal(2′) residue in KAZO_5.3 resulted in a distinct binding mode despite maintaining a highly favorable docking score (Table 1, Figure 2C). The ligand retained several hydrogen-bond interactions involving SER222, LYS141, PHE204 and THR207, indicating preserved receptor recognition capability. Nevertheless, the enlarged aromatic naphthyl system significantly altered ligand geometry and produced a more sterically crowded binding mode. Compared with KAZO_5.2, the peptide adopted a less compact conformation and lost several stabilizing central interactions, particularly those associated with GLU310 and ALA304 (Figure 2B,C). These observations suggest that excessive aromatic bulk may negatively affect optimal accommodation within the MOR binding pocket. Consequently, although Nal(2′) increased hydrophobic character, it likely introduced steric constraints that resulted in less favorable receptor accommodation in the docking model.

The unfavorable structural consequences of excessive ligand elongation became even more pronounced for KAZO_7.1. This peptide adopted a highly extended conformation spanning a large portion of the receptor surface. Multiple hydrogen bonds involving GLY52, SER222, THR225, and LYS209 were observed; however, the ligand lacked compact packing within the central receptor cavity (Table 1, Figure 2D). The absence of Dmt and D-Phe residues likely contributed to reduced anchoring efficiency of the N-terminal opioid pharmacophore. Moreover, the elongated peptide structure probably increased the entropic cost of receptor binding and promoted higher conformational mobility. These features collectively suggest less favorable receptor accommodation compared with the Dmt-containing analogues. Interestingly, incorporation of Dmt and D-Phe into the longer peptide scaffold generated KAZO_7.2, which displayed the most favorable docking score within the entire investigated series (GlideScore = −13.184; Table 1). The ligand established multiple stabilizing hydrogen bonds involving GLY52, PHE221, THR225, ASN230, and ALA304 (Figure 2E). In contrast to KAZO_7.1, the peptide adopted a considerably more compact conformation despite its larger size. Both the central and C-terminal fragments actively contributed to receptor stabilization through interactions with residues located in deeper regions of the binding cavity. These findings indicate that Dmt and D-Phe residues effectively compensated for the unfavorable conformational effects associated with peptide elongation. A similar trend was observed for KAZO_7.3 (Table 1, Figure 2F), in which D-Phe was replaced by Nal(2′). The ligand preserved several favorable interactions involving THR160 and ASN191, indicating efficient receptor recognition. However, the bulky naphthyl group again appeared to negatively affect ligand compactness and overall geometric organization within the receptor cavity. Compared with KAZO_7.2, the peptide adopted a more extended binding mode and likely exhibited increased conformational flexibility.

As for the interaction with D2R, all investigated ligands yielded favorable GlideScores, ranging from −14.673 to −15.987 (Table 1). These scores were used to compare relative docking favorability within the KAZO series rather than as quantitative estimates of receptor binding affinity. Notably, the differences between individual analogues were less pronounced than those observed for MOR, suggesting that the D2R binding pocket may accommodate a broader range of peptide conformations and structural modifications.

**Figure 3:**
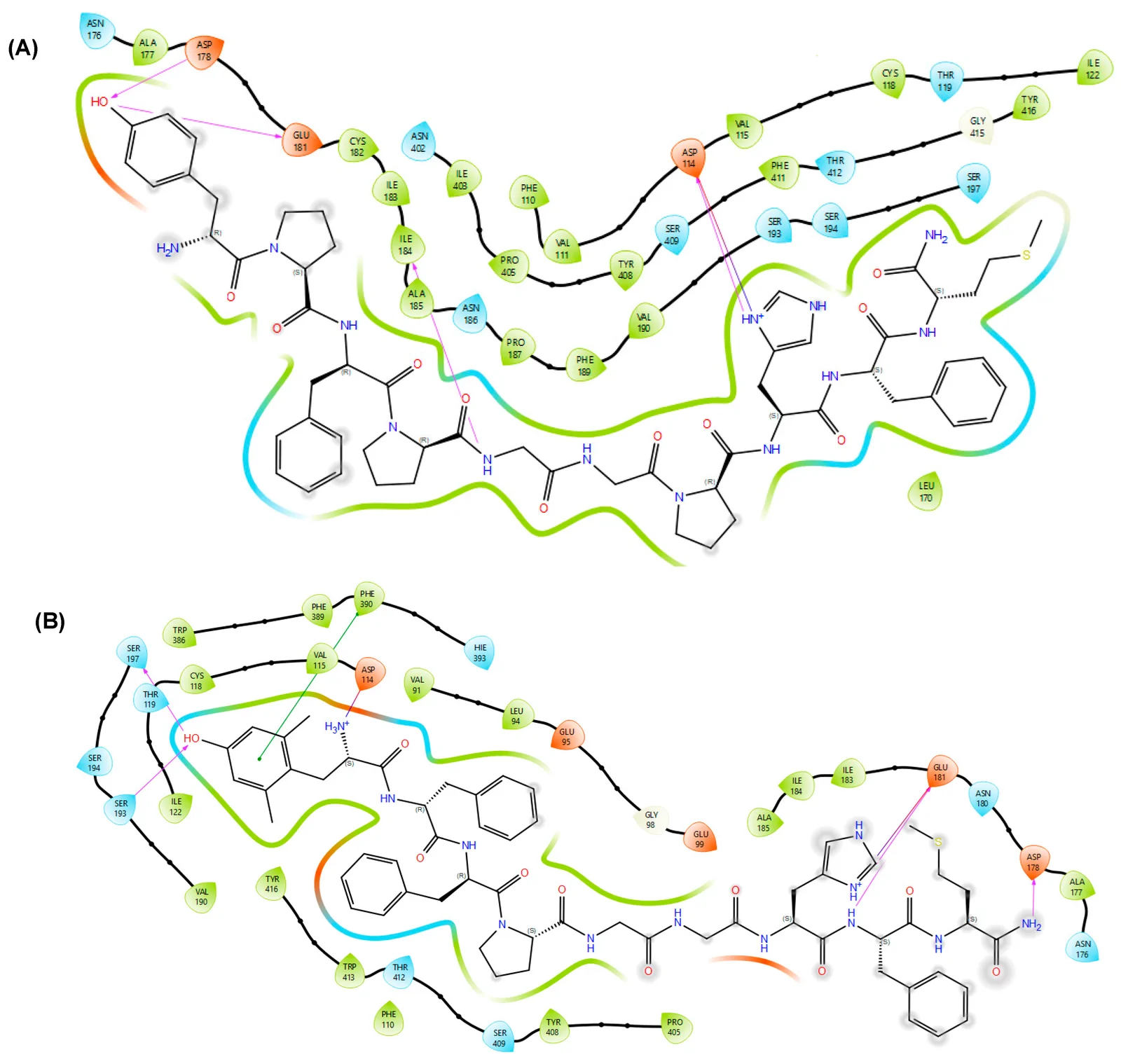

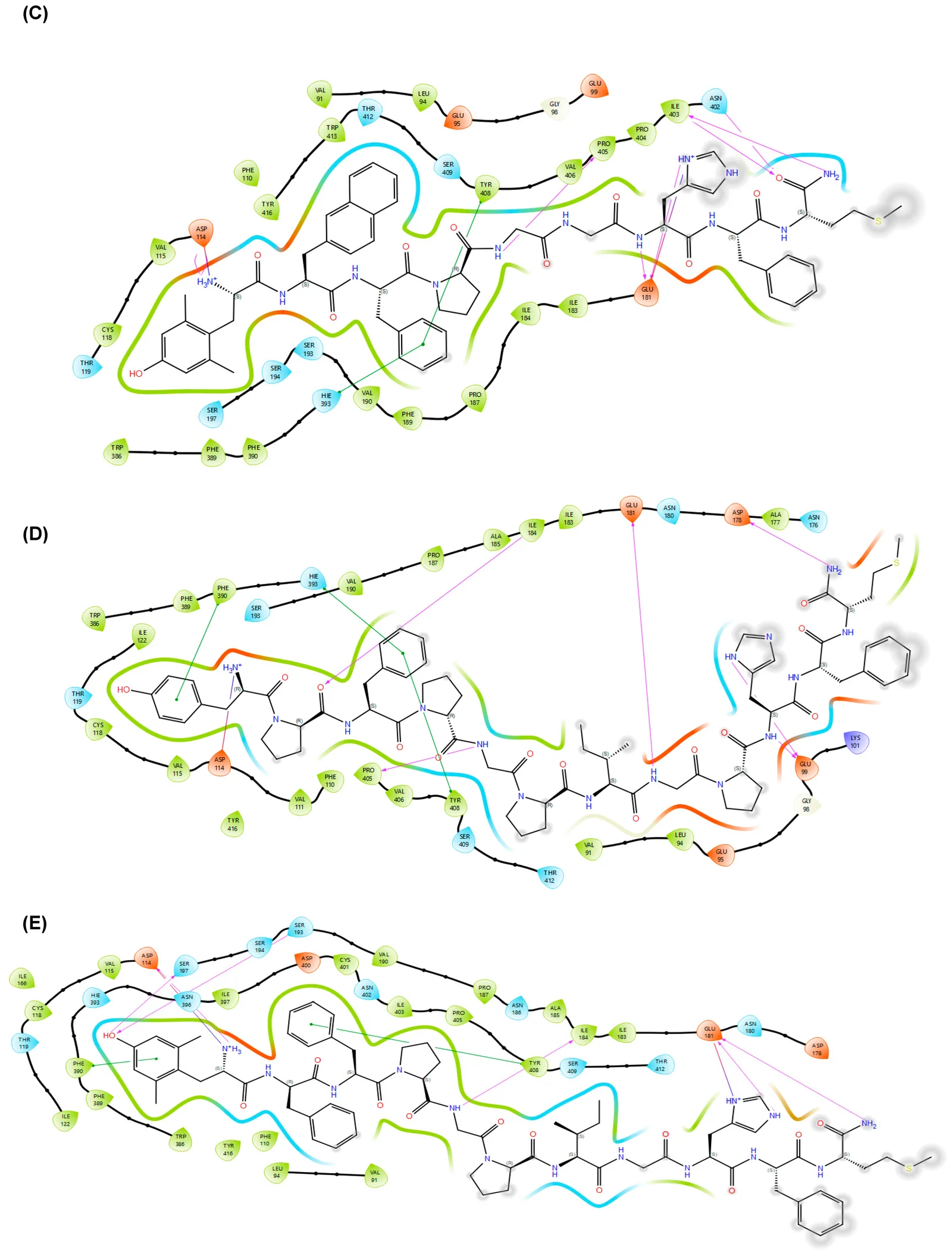

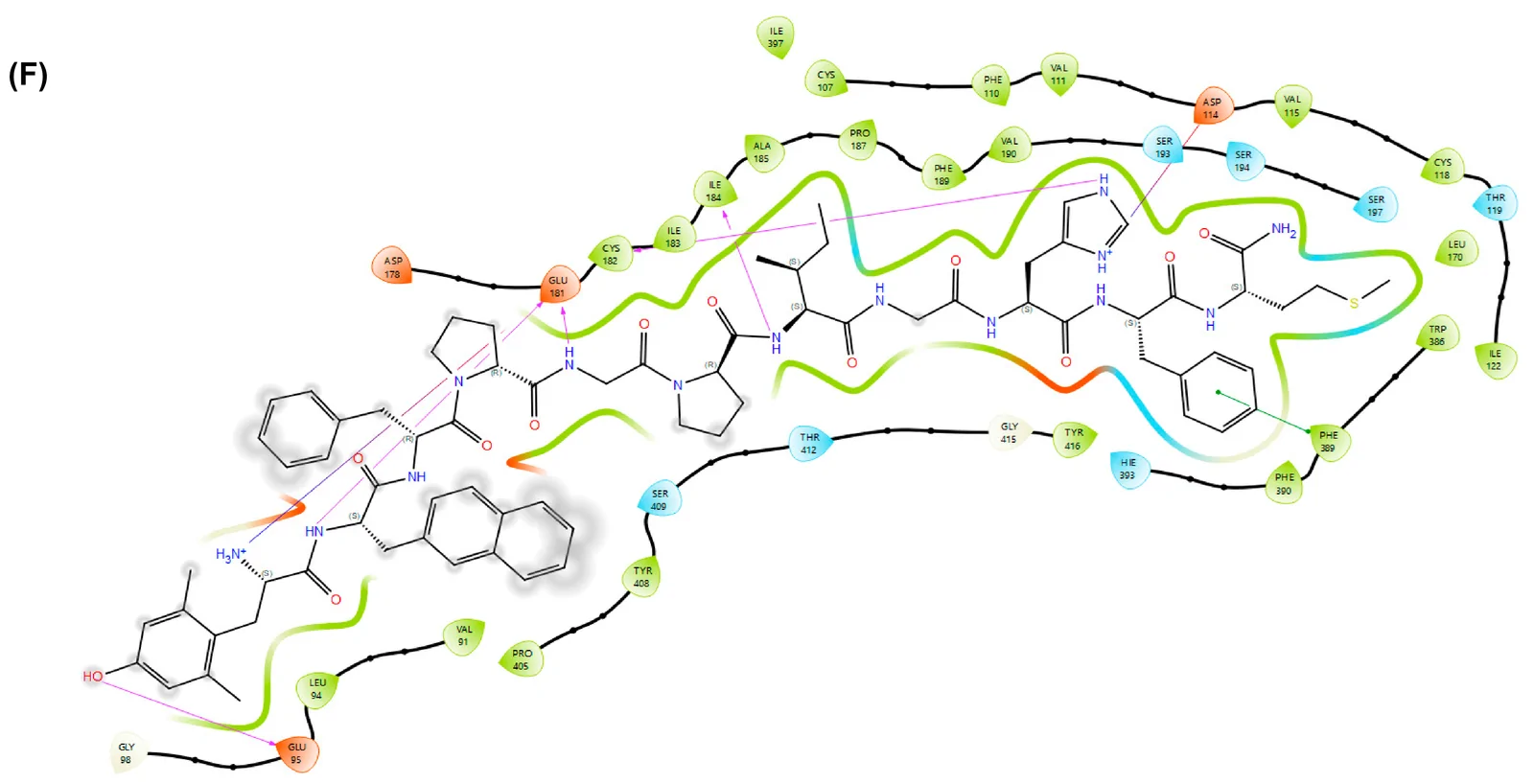
Two-dimensional ligand–receptor interaction diagrams for the investigated peptides in complex with the human dopamine D2 receptor (D2R, PDB ID: 7JVR) obtained using Glide SP-Peptide docking. Hydrogen bonds are represented by purple arrows, while π-type interactions are shown as red arrows. Hydrophobic interactions and surrounding receptor residues contributing to ligand stabilization within the binding pocket are also indicated. (**A**) KAZO_5.1; (**B**) KAZO_5.2; (**C**) KAZO_5.3; (**D**) KAZO_7.1; (**E**) KAZO_7.2; (**F**) KAZO_7.3.

Docking-pose analysis identified several recurring interactions conserved across the investigated series. ASP114 and GLU181 repeatedly formed hydrogen bonds and appeared central to ligand recognition. ASP114 interacted with KAZO_5.1, KAZO_5.2, KAZO_5.3, KAZO_7.1, and KAZO_7.2, indicating its role as a potential anchoring residue for peptide binding. Similarly, GLU181 stabilized most complexes, particularly those containing Dmt-substituted analogues (Figure 3). The shortest analogue, KAZO_5.1, formed hydrogen bonds with ASP178, GLU181, ILE184, and ASP114, and was further stabilized by extensive hydrophobic contacts. This ligand occupied a substantial part of the orthosteric cavity and interacted through both its N- and C-terminal regions (Figure 3A). In KAZO_5.2, introduction of Dmt and D-Phe produced a more compact binding mode and increased the contribution of aromatic and hydrophobic residues surrounding the N-terminal pharmacophore. In addition to interactions with ASP114 and GLU181, this ligand contacted SER193, SER197, and ASP178. The Dmt-D-Phe-Phe motif occupied a highly hydrophobic receptor region, suggesting that aromatic packing substantially contributes to complex stabilization (Table 1, Figure 3B). KAZO_5.3, in which D-Phe was replaced by Nal(2′), showed a similar binding pattern (Table 1, Figure 3C). The bulky naphthyl group was efficiently accommodated within the receptor cavity and formed extensive contacts with residues such as HIE393, TYR408, and PRO405. Unlike in MOR, where Nal(2′) appeared to impose steric constraints, the D2R binding pocket accommodated the larger aromatic group without markedly disrupting the overall binding geometry (Figure 2C and Figure 3C).

The longer analogues KAZO_7.1–7.3 displayed more extended conformations (Figure 3D–F); however, they remained well accommodated within the receptor cavity. KAZO_7.1 retained key interactions with ASP114, ILE184, GLU181, ASP178, and GLU99 (Figure 3D), indicating that elongation of the peptide chain did not prevent productive receptor recognition. This observation differs markedly from the behavior observed in MOR (Figure 2D), where peptide elongation was associated with less favorable binding geometries. Among the longer analogues, KAZO_7.2 exhibited the most favorable docking score (GlideScore = −15.987; Table 1). The ligand formed hydrogen bonds with ASP114, SER193, SER197, and GLU181 while simultaneously engaging an extensive hydrophobic pocket composed of TRP386, PHE389, PHE390, ILE397, LEU403, and PRO405 (Figure 3E). The compact organization of the Dmt-D-Phe-containing pharmacophore and the large number of stabilizing contacts suggest particularly efficient receptor accommodation. Finally, KAZO_7.3 combined the elongated peptide scaffold with the Nal(2′) residue. The ligand established interactions with GLU95, GLU181, CYS182 and ILE184 while maintaining extensive hydrophobic contacts with TRP386, PHE389, PHE390, TYR408, and TYR416 (Figure 3F). As observed for KAZO_5.3, the naphthyl substituent was readily accommodated within the hydrophobic region of the receptor, further supporting the notion that D2R possesses a greater tolerance toward bulky aromatic fragments than MOR.

Overall, the docking results demonstrate that ligand recognition by MOR and D2R is governed by distinct structural requirements despite several common interaction patterns. In the MOR system, ligand compactness and optimal aromatic substitution within the N-terminal pharmacophoric region emerged as key determinants of receptor recognition and docking performance. Introduction of Dmt and D-Phe residues consistently enhanced ligand accommodation within the binding pocket and promoted the formation of stabilizing interactions, whereas substitution with the bulkier Nal(2′) residue generally resulted in less favorable docking outcomes despite preserving several favorable receptor contacts. These observations suggest that the MOR binding pocket favors a balanced combination of conformational rigidity, steric compatibility, and appropriately positioned aromatic interactions rather than maximal aromatic expansion. In contrast, D2R recognition was primarily governed by hydrogen-bond interactions involving ASP114 and GLU181 together with extensive hydrophobic contacts formed within an aromatic-rich binding cavity. The recurrent participation of residues TRP386, PHE389, PHE390, TYR408, and TYR416 highlights the importance of aromatic and hydrophobic interactions in stabilizing the ligand–receptor complexes. Furthermore, the ability of D2R to accommodate both elongated peptide structures and bulky Nal(2′)-containing analogues indicates substantial conformational flexibility of the receptor binding pocket. These features likely contribute to the consistently favorable docking scores observed for the entire KAZO series at D2R. Collectively, the results reveal notable differences in ligand recognition mechanisms between MOR and D2R, with MOR exhibiting greater sensitivity to ligand geometry and steric effects, whereas D2R appears more tolerant toward structural elongation and increased aromatic bulk.

### 2.2. Effects of Hybrid Peptides on Cell Viability

**Figure 4:**
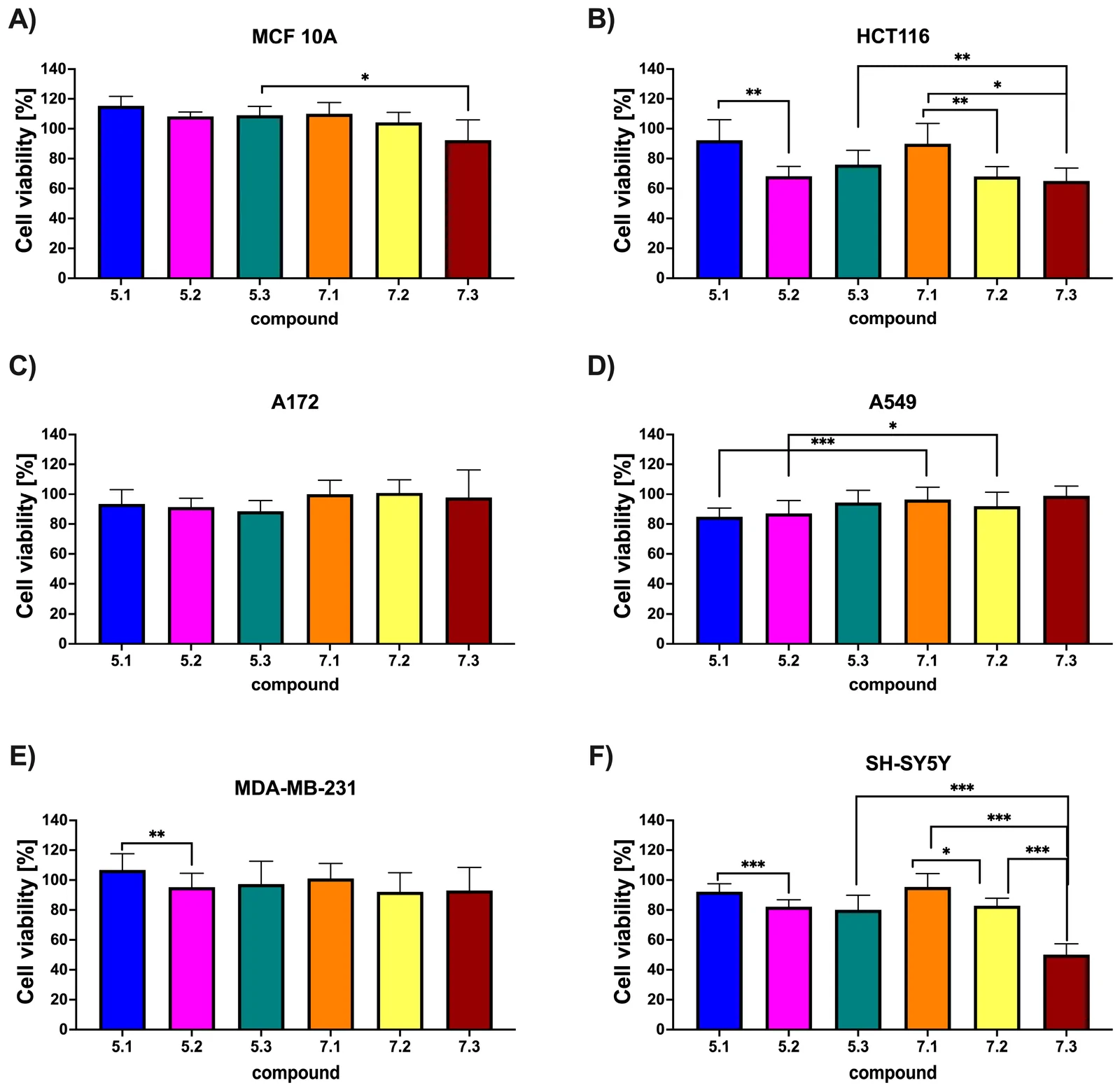
Cell viability assessed by the MTT assay in different cell lines treated with hybrid compounds from the KAZO_5 and KAZO_7 series. (**A**) MCF 10A (non-tumorigenic control), (**B**) HCT116, (**C**) A-172, (**D**) A549, (**E**) MDA-MB-231, and (**F**) SH-SY5Y cells were treated with six compounds, as indicated. Each panel shows the response of a single cell line to all tested compounds at a single concentration of 300 µg/mL for 48 h. Experiments were performed three independent times, each with multiple technical replicates. Data are presented as mean ± SD. Statistical analysis was performed using one-way ANOVA followed by Tukey’s multiple comparisons test for HCT116 cells, while a mixed-effects model with Tukey’s post-hoc test was applied for the remaining cell lines to account for missing values. Statistical significance is indicated as *p* < 0.05 (*), 0.001 ≤ *p* < 0.01 (**), and *p* < 0.001 (***).

Initial screening of the KAZO_5 and KAZO_7 hybrid series across multiple cancer cell lines revealed compound- and cell line-dependent effects on viability (Figure 4). Among the tested peptides, KAZO_5.2, KAZO_7.2, and KAZO_7.3 produced the most pronounced viability-reducing effects, particularly in HCT116 cells (KAZO_5.2: 68.2 ± 6.5%; KAZO_7.2: 68.0 ± 6.6%; KAZO_7.3: 65.7 ± 8.6%; Figure 4B) and SH-SY5Y (KAZO_7.3: 50.2 ± 7.2%; Figure 4F), whereas the remaining analogs demonstrated moderate or negligible activity.

Notably, although KAZO_7.3 produced the strongest apparent viability-reducing effect at 300 µg/mL (*p* < 0.001), particularly in SH-SY5Y cells (Figure 4F), further investigation of this compound was not pursued. Stability studies, including LC-MS analysis in culture medium (Figure S2), demonstrated substantial degradation of KAZO_7.3 under these conditions. Consequently, the observed antiproliferative activity is most likely attributable to degradation products rather than the intact parent chimera. Based on these findings, KAZO_7.3 was excluded from further mechanistic analyses, while both KAZO_5.2 and KAZO_7.2 were selected for further studies in HCT116 cells.

**Figure 5:**
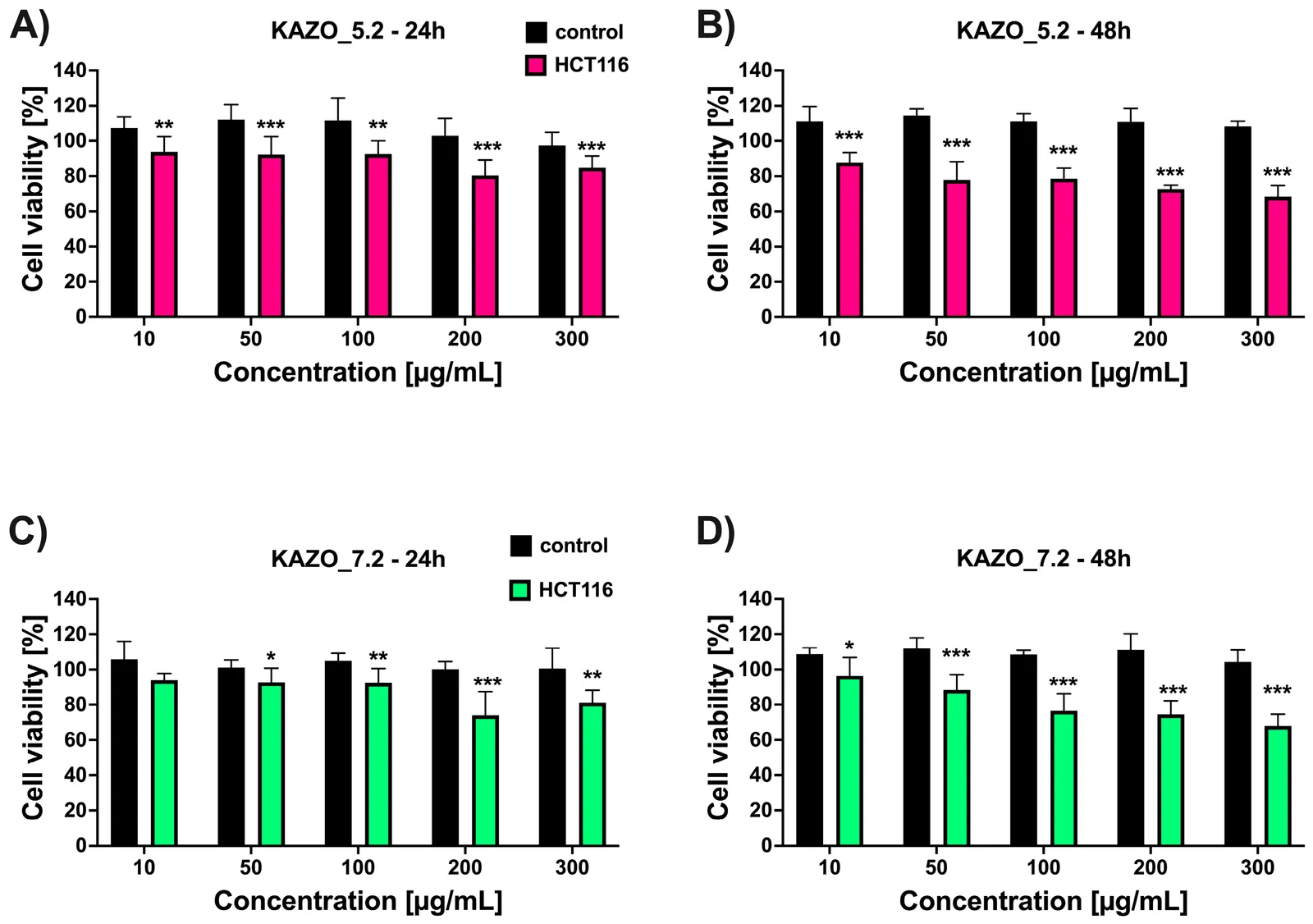
Dose- and time-dependent effects of KAZO_5.2 and KAZO_7.2 on cell viability assessed by the MTT assay. HCT116 cells were treated with increasing concentrations of each compound (10, 50, 100, 200, and 300 µg/mL) for 24 h or 48 h. Non-tumorigenic MCF 10A cells treated with the same compounds served as the control. Panels show: (**A**) KAZO_5.2, 24 h; (**B**) KAZO_5.2, 48 h; (**C**) KAZO_7.2, 24 h; (**D**) KAZO_7.2, 48 h. Experiments were performed three independent times with multiple technical replicates; some data points were missing. Data are presented as mean ± SD. Statistical analysis was performed using a mixed-effects model to account for missing values, with Sídák’s post-hoc test for multiple comparisons. *p* < 0.05 (*), 0.001 ≤ *p* < 0.01 (**), *p* < 0.001 (***).

Detailed analysis confirmed that both peptides reduced HCT116 viability in a dose- and time-dependent manner (Figure 5). For example, after 48 h, treatment with 200 µg/mL and 300 µg/mL decreased cell viability to approximately 72.6 ± 2.2% and 68.2 ± 6.5% (KAZO_5.2; *p* < 0.001; Figure 5B) and 74.5 ± 7.7% and 68.0 ± 6.6% (KAZO_7.2; *p* < 0.001; Figure 5D), respectively. Notably, neither compound significantly affected the viability of MCF 10A cells (Figure 5). For reference, based on the molecular weights of KAZO_5.2 (1127.9 g/mol) and KAZO_7.2 (1388.1 g/mol), the 200 and 300 µg/mL concentrations used in these experiments correspond to approximately 177.4 and 266.0 µM (KAZO_5.2), and 144.1 and 216.1 µM (KAZO_7.2), respectively. Nevertheless, under the tested conditions, IC50 values could not be determined for either compound, as a 50% reduction in cell viability was not achieved within the tested concentration range.

As a complementary measure of selectivity, a selectivity ratio (SR = %viability HCT116/%viability MCF 10A) was calculated at 200 and 300 µg/mL after 24 h and 48 h. For KAZO_5.2, SR values decreased from 0.780 to 0.656 (200 µg/mL) and from 0.870 to 0.630 (300 µg/mL) between 24 h and 48 h. A similar trend was observed for KAZO_7.2, with SR decreasing from 0.739 to 0.672 (200 µg/mL) and from 0.807 to 0.652 (300 µg/mL). Notably, the concentration-dependence of SR was less consistent than its time-dependence: at 24 h, SR was higher at 300 µg/mL than at 200 µg/mL for both compounds, whereas at 48 h this relationship was reversed. These findings indicate that both compounds exhibited enhanced selectivity toward HCT116 cells relative to the non-tumorigenic MCF 10A line with prolonged incubation, consistent with the time-dependent (but not consistently concentration-dependent) activity described above. This pattern suggests that the observed selectivity margin may be more strongly associated with duration of exposure than with compound concentration within the tested range, potentially reflecting a cumulative, time-dependent pharmacodynamic effect; however, this observation should be interpreted with caution and warrants further investigation.

As both KAZO_5.2 and KAZO_7.2 exhibited comparable viability-reducing effects in HCT116 cells at 200 µg/mL (i.e., 177.4 µM and 144.1 µM, respectively) and 300 µg/mL (i.e., 266 µM and 216.1 µM, respectively) after 48 h (Figure 5), further mechanistic studies were conducted at 200 µg/mL to minimize potential non-specific toxicity associated with higher concentrations.

### 2.3. Effect of Naloxone Pretreatment on the Viability-Reducing Effect of Selected Hybrid Peptides in HCT116 Cells

**Figure 6:**
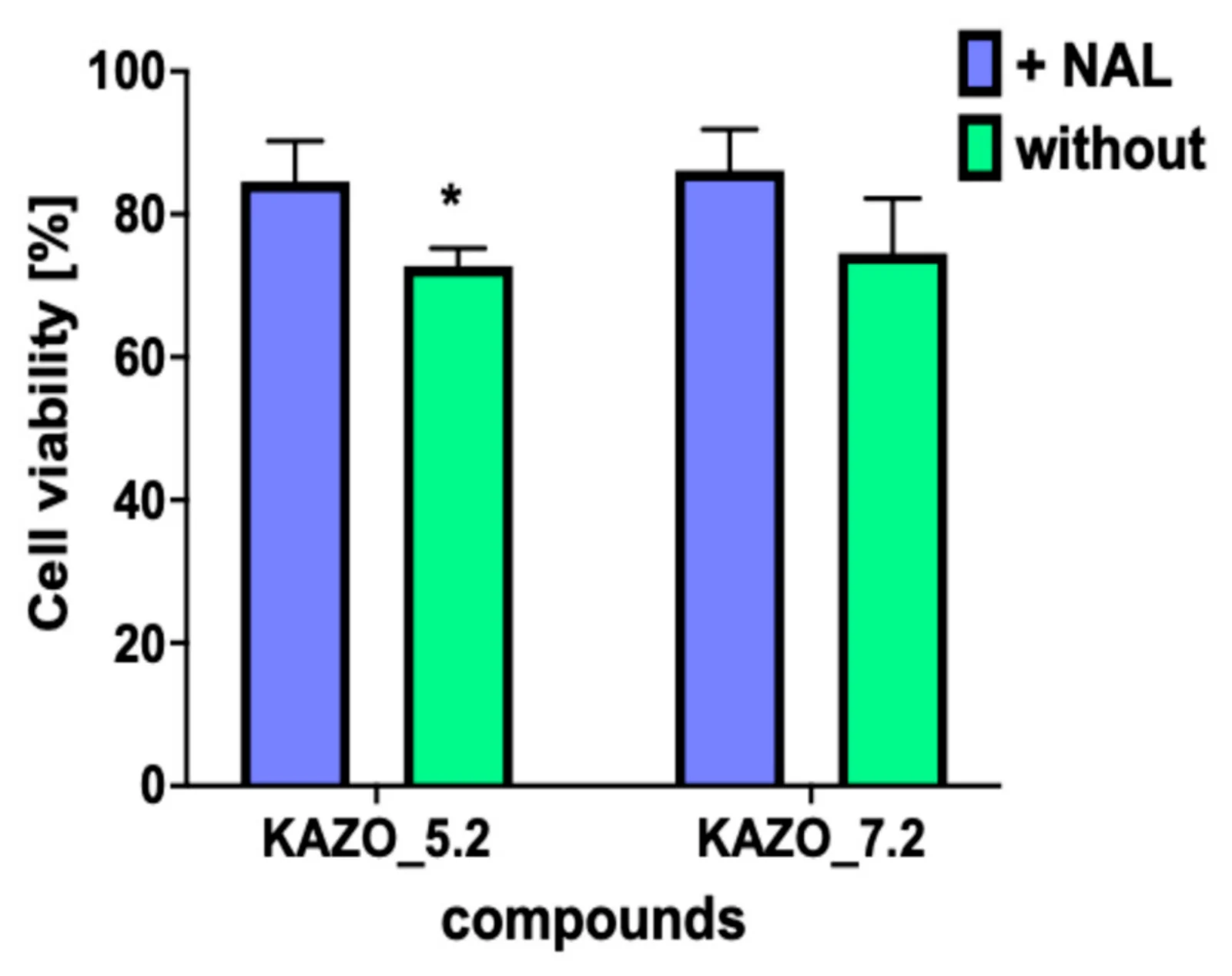
Effect of naloxone (NAL) pretreatment on the reduction in cell viability induced by KAZO_5.2 and KAZO_7.2 at 200 µg/mL. Cells were pretreated with NAL prior to chimera administration. Cell viability is presented as mean ± SD. Data were analyzed by two-way ANOVA with Bonferroni’s multiple comparisons test. NAL significantly increased cell viability for KAZO_5.2 (* *p* < 0.05). For KAZO_7.2, the increase did not reach statistical significance.

To evaluate the contribution of MOR-related mechanisms to the viability-reducing effects of the hybrid compounds, HCT116 cells were pretreated with naloxone prior to administration of the chimeras at 200 µg/mL. For KAZO_5.2, cell viability increased from 72.74 ± 2.49% to 84.56 ± 5.68% in the presence of NAL, indicating a modest attenuation of the biological effect. Similarly, pretreatment with NAL increased cell viability in KAZO_7.2-treated cells from 74.53 ± 7.68% to 86.12 ± 5.73% (Figure 6). The increase in cell viability following NAL pretreatment reached statistical significance for KAZO_5.2 (*p* < 0.05), whereas for KAZO_7.2, the observed increase did not reach statistical significance (*p* > 0.05; Figure 6).

This incomplete reversal of activity observed for both compounds suggests that opioid receptor signaling pathways may contribute to their biological activity but are unlikely to fully explain the mechanism responsible for the observed reduction in cell viability. The partial antagonistic effect of NAL may also reflect the multifunctional nature of the synthesized hybrid peptides. In addition, molecular docking and molecular dynamics analyses yielded favorable predicted interaction modes and receptor-associated trajectories for D2R (Figure 2 and Figure 3), providing a structural hypothesis for possible D2R engagement rather than direct evidence of receptor binding. Therefore, the observed biological activity of KAZO_5.2 and KAZO_7.2 may result from the simultaneous modulation of multiple receptor-mediated signaling pathways rather than selective MOR activation alone. This possibility is further supported by the fact that the GHFM pharmacophore incorporated into the present hybrids was derived from LENART01, a structurally related opioid-dopaminergic chimera for which functional D2R binding activity has been experimentally confirmed [23], lending indirect support to the plausibility of D2R engagement by the KAZO series. Nevertheless, pharmacological blockade of D2R was not performed in the present study, and D2R expression in HCT116 cells was not verified; direct functional confirmation of D2R involvement, together with assessment of receptor expression, therefore remains an important objective for future investigations.

Interpretation of the NAL experiments is further complicated by the inconsistent literature regarding MOR expression in HCT116 cells. Several studies have reported MOR/OPRM1 expression in colorectal cancer tissues and have classified HCT116 cells as MOR-positive [25,26,27]; however, reported expression levels vary considerably between studies and experimental models. Consistent with this variability, only a faint MOR/OPRM1 signal was detected in HCT116 cells under our experimental conditions using Western blot analysis (Figure S3), suggesting low baseline receptor expression; formal densitometric quantification was not performed, and therefore potential treatment-related changes in receptor expression could not be assessed. Nevertheless, this discrepancy may reflect methodological differences as well as the technical challenges associated with the detection of endogenous GPCRs, including low receptor abundance, antibody-related limitations, receptor glycosylation, and sensitivity to sample preparation procedures [28,29,30]. Therefore, although the present results suggest that opioid-sensitive pathways may contribute to the biological activity of the investigated hybrids, definitive conclusions regarding direct MOR involvement cannot be drawn at this stage.

### 2.4. Cell-Cycle Distribution and Apoptosis Analysis Following Treatment with Selected Hybrid Peptides

**Figure 7:**
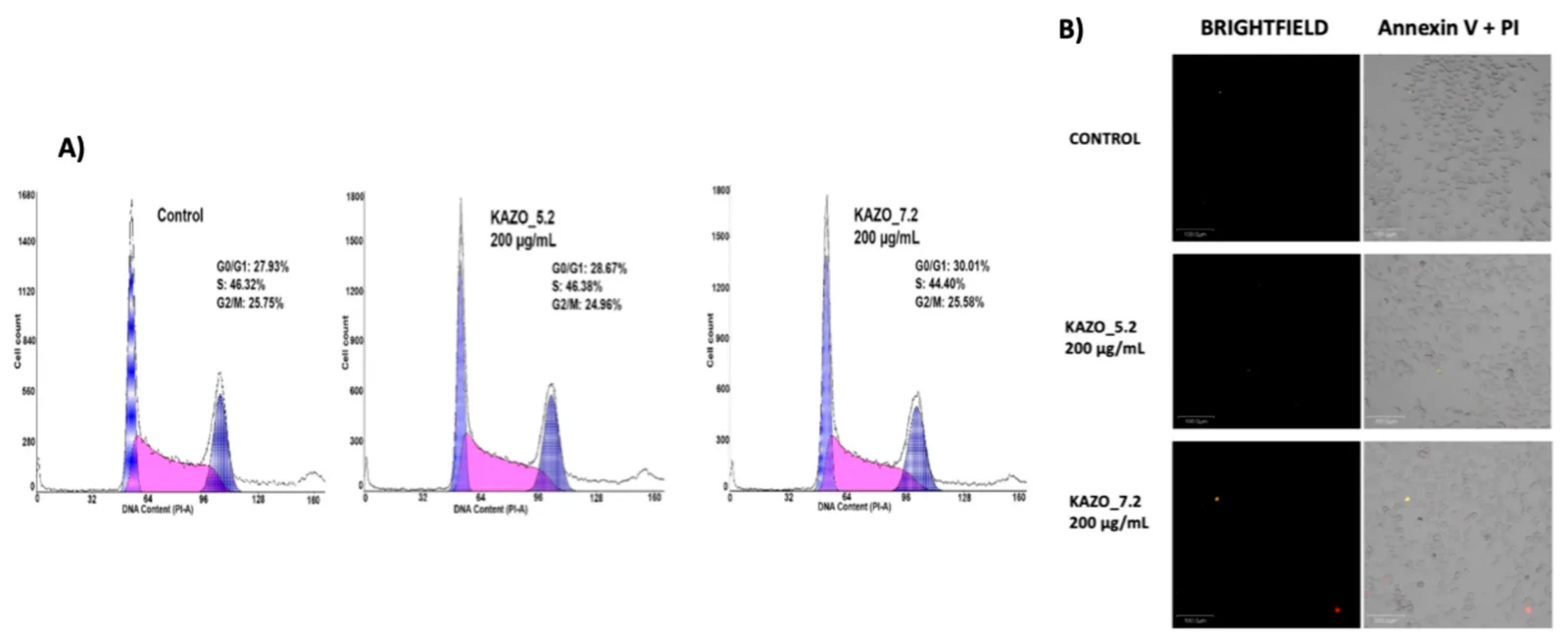
Cell cycle distribution (**A**) and representative Annexin V-FITC/PI staining (**B**) of HCT116 cells after 48 h treatment with KAZO_5.2 and KAZO_7.2 (200 µg/mL). DNA content was analyzed by flow cytometry following propidium iodide (PI) staining. Representative histograms show G0/G1, S, and G2/M phases, with percentages indicated in each panel. Annexin V (green) and PI (red). Images acquired at 10× magnification. Scale bar: 100 µm. Images are representative of qualitative assessment only; no formal quantification (e.g., cell counting or flow cytometric quantification of Annexin V-/PI-positive populations) was performed, and therefore, no statistical comparison is presented for this panel.

Cell cycle distribution was analyzed in HCT116 cells following 48 h treatment with KAZO_5.2 and KAZO_7.2 at 200 µg/mL (177.4 µM and 144.1 µM, respectively) (Figure 7A). Compared to control cells, neither compound induced a pronounced accumulation of cells in any specific phase of the cell cycle. The distribution of cells across G0/G1, S, and G2/M phases remained relatively similar between treated and control groups. These results suggest that the reduction in metabolic activity mediated by KAZO_5.2 and KAZO_7.2 is not associated with a strong cell cycle arrest under the tested conditions.

Similarly, no evident increase in Annexin V- or PI-positive cells was observed in treated samples compared to control (Figure 7B), indicating that, based on the qualitative observations, the tested compounds did not induce pronounced apoptosis under the applied conditions.

Taken together, these findings indicate that the reduction in MTT-derived viability observed after treatment with KAZO_5.2 and KAZO_7.2 is not accompanied by substantial induction of apoptosis or marked perturbation of cell-cycle progression. Therefore, under the tested conditions, the observed effect may primarily reflect suppression of cellular metabolic activity and/or proliferation rather than classical cytotoxicity. Further studies will be required to determine the precise mechanisms responsible for the reduced viability signal.

### 2.5. Hemolytic Activity of KAZO_5.2 and KAZO_7.2 Hybrid Peptides

**Figure 8:**
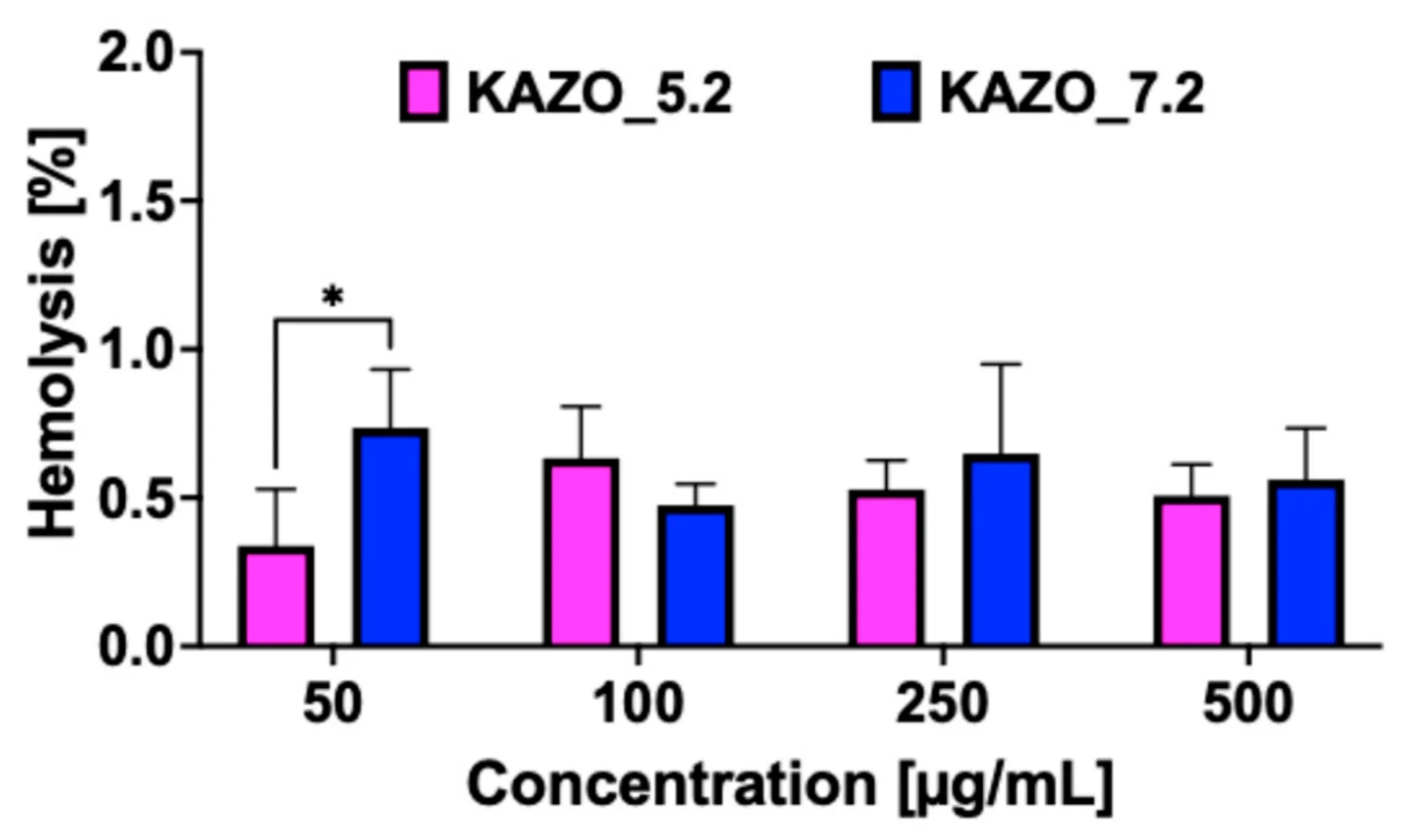
Dose-dependent hemolytic activity of KAZO_5.2 and KAZO_7.2 chimeras after 1 h of incubation. Two-way ANOVA with Bonferroni’s post-hoc test revealed significant differences between both compounds at a concentration of 50 µg/mL; *p* < 0.05 (*).

As shown in Figure 8, both tested compounds exhibited negligible hemolytic activity, with the maximal hemolysis remaining below 2% across the tested concentration range. Importantly, the shorter amino acid sequence of KAZO_5.2 compared to KAZO_7.2 did not substantially affect its interaction with erythrocytes at most concentrations. A statistically significant difference between the two peptides was observed only at 50 µg/mL (*p* < 0.05), where KAZO_5.2 demonstrated a slightly more favorable safety profile.

### 2.6. Enzymatic Stability of KAZO_5.2 and KAZO_7.2 Peptides

**Figure 9:**
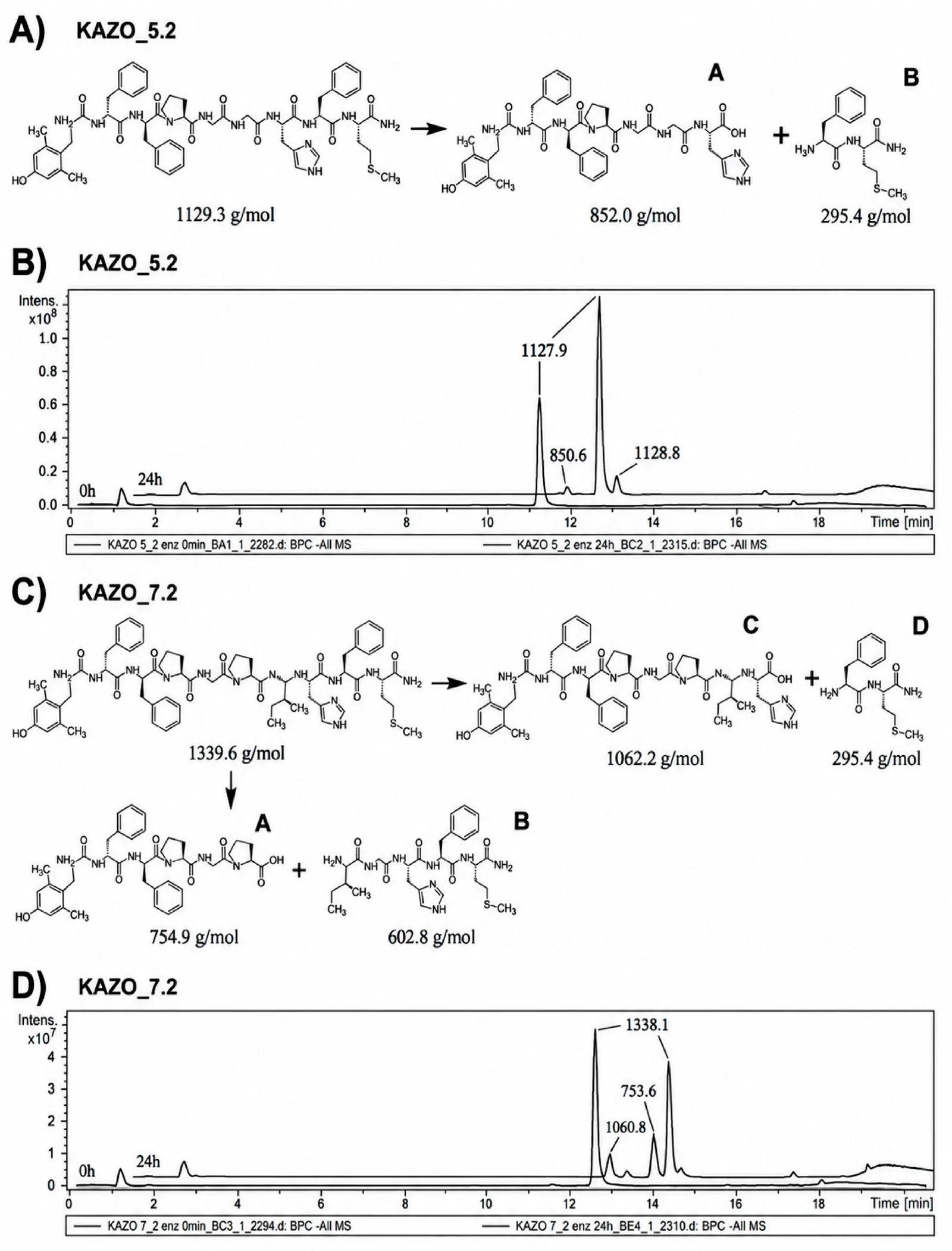
Representative LC-MS analysis of KAZO peptides after incubation in human plasma. (**A**) Proposed degradation products of KAZO_5.2 (*m*/*z* = 1127.9) identified based on the observed molecular weights: Dmt-(D-Phe)-FPGGH (*m*/*z* = 850.6); (**B**) Representative LC-MS chromatograms of KAZO_5.2 recorded at 0 h and after 24 h incubation at 37 °C. (**C**) Proposed degradation products of KAZO_7.2 (*m*/*z* = 1388.1) identified based on the observed molecular weights: Dmt-(D-Phe)-FPGP (*m*/*z* = 753.6) and Dmt-(D-Phe)-FPGPIGH (*m*/*z* = 1060.8). (**D**) Representative LC-MS chromatograms of KAZO_7.2 recorded at 0 h and after 24 h incubation at 37 °C. Structures termed as “A” and “C” are experimentally observed degradation product, while “B” and “D” are putative degradation products.

Incubation of KAZO_5.2 and KAZO_7.2 in human plasma for 24 h at 37 °C resulted in only limited degradation of both peptides. Representative LC-MS chromatograms (Figure 9B,D) showed that the intact peptides remained the predominant species after 24 h of incubation, whereas only minor degradation products were detected. The proposed degradation products, identified based on their observed molecular masses (Figure 9A,C), indicate cleavage at only a limited number of peptide bonds, with the N-terminal Dmt-(D-Phe)-containing fragment consistently retained as a stable, proteolytically resistant core. The high resistance of both peptides to enzymatic degradation can be attributed to the presence of non-proteinogenic residues at the N-terminus, namely Dmt (2′,6′-dimethyltyrosine) and D-Phe, which are known to sterically hinder recognition and cleavage by plasma peptidases, thereby significantly enhancing metabolic stability [31,32,33,34,35]. These results suggest that the apparent half-lives of both peptides in human plasma likely exceed 24 h, which compares favorably with the stability typically reported for unmodified linear peptides, where half-lives often range from minutes to a few hours [36] and indicates a potentially favorable pharmacokinetic profile for both compounds.

### 2.7. Molecular Dynamics Simulation of Selected KAZO Peptides in Complex with MOR and D2R

**Figure 10:**
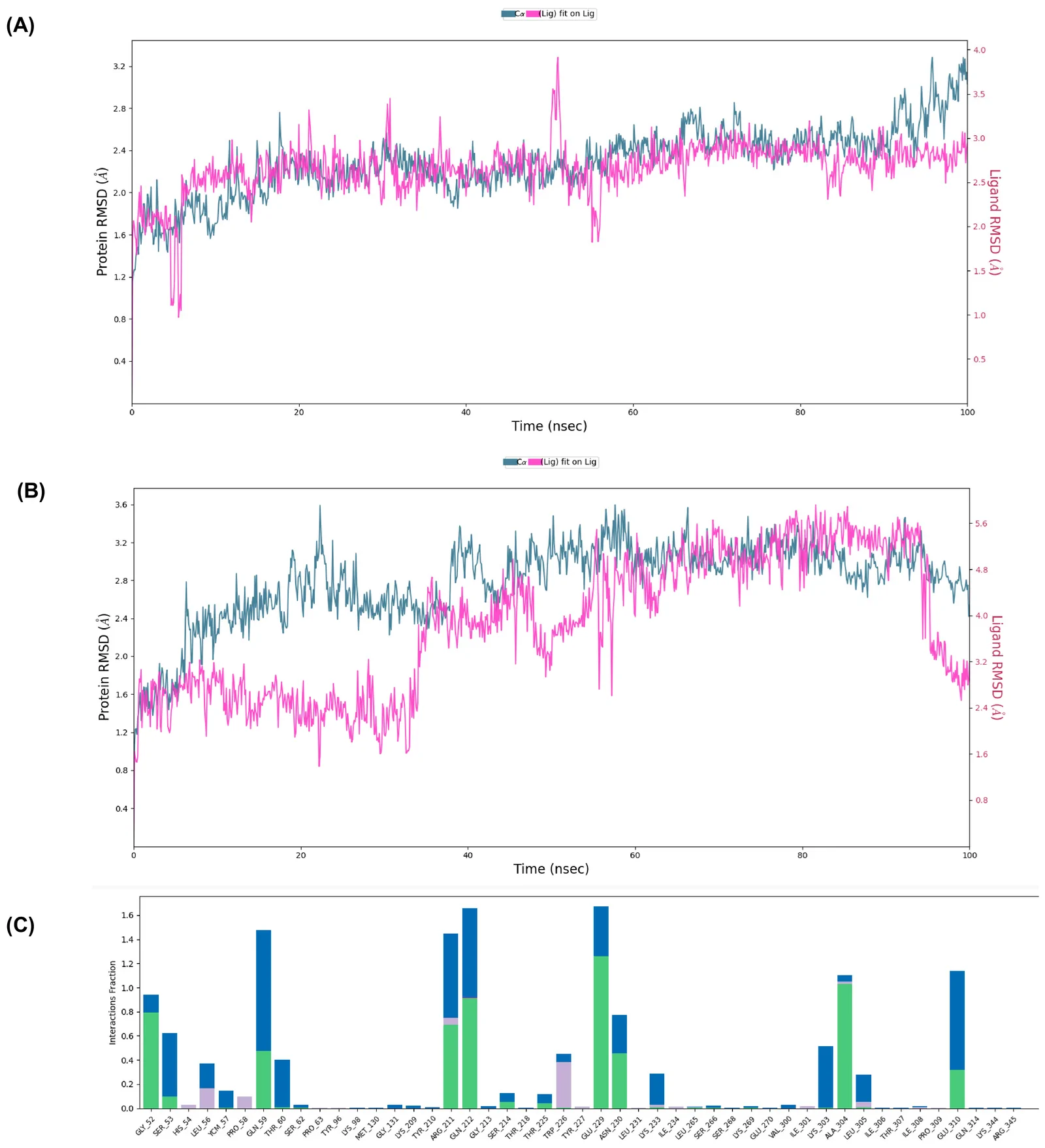

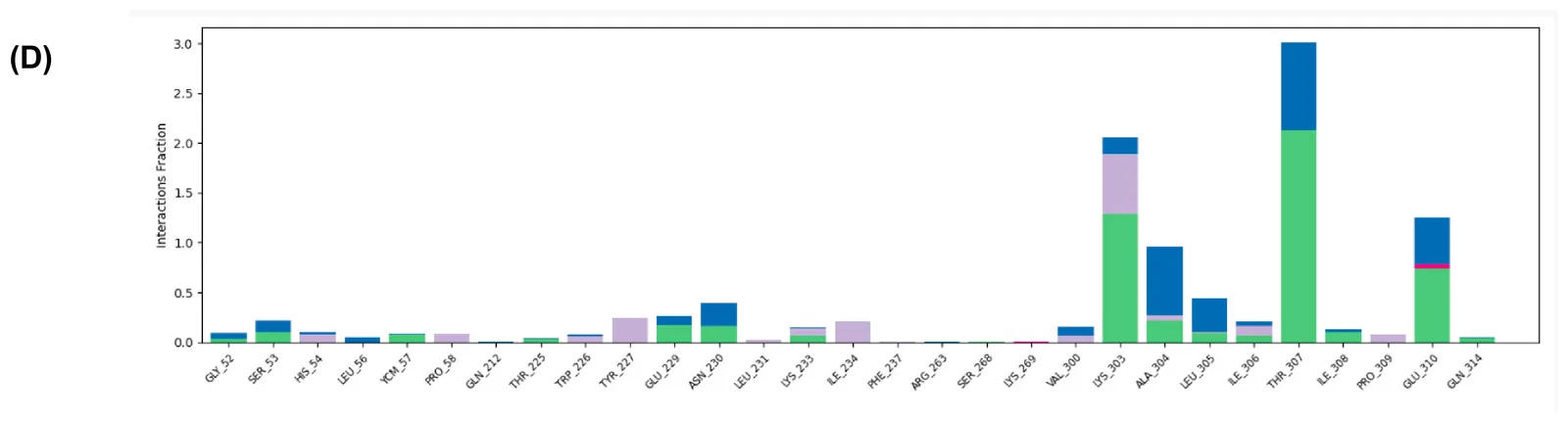
Root-mean-square deviation (RMSD) plots and protein–ligand interaction profiles obtained from 100 ns molecular dynamics simulations of the investigated peptide analogues in complex with the μ-opioid receptor (MOR). Protein Cα RMSD values are shown in blue, while ligand RMSD values are shown in magenta. Protein–ligand contact histograms present the fraction of simulation time during which individual receptor residues interacted with the ligand. Interactions are classified as hydrogen bonds (green), hydrophobic contacts (purple), ionic interactions (red), and water bridges (blue). (**A**) RMSD analysis for the MOR–KAZO_5.2 complex; (**B**) RMSD analysis for the MOR–KAZO_7.2 complex; (**C**) protein–ligand contacts for the MOR–KAZO_5.2 complex; and (**D**) protein–ligand contacts for the MOR–KAZO_7.2 complex.

**Figure 11:**
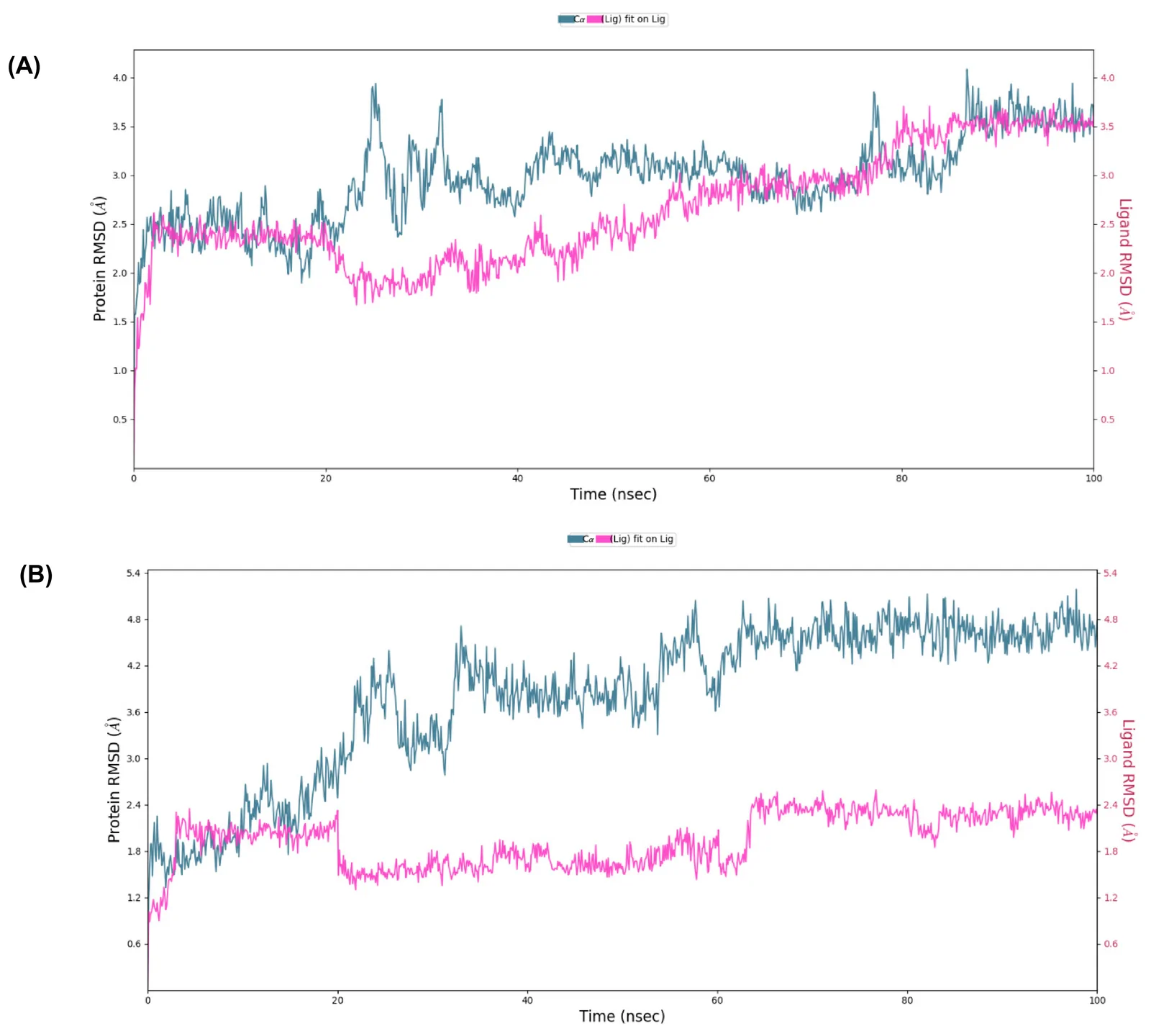

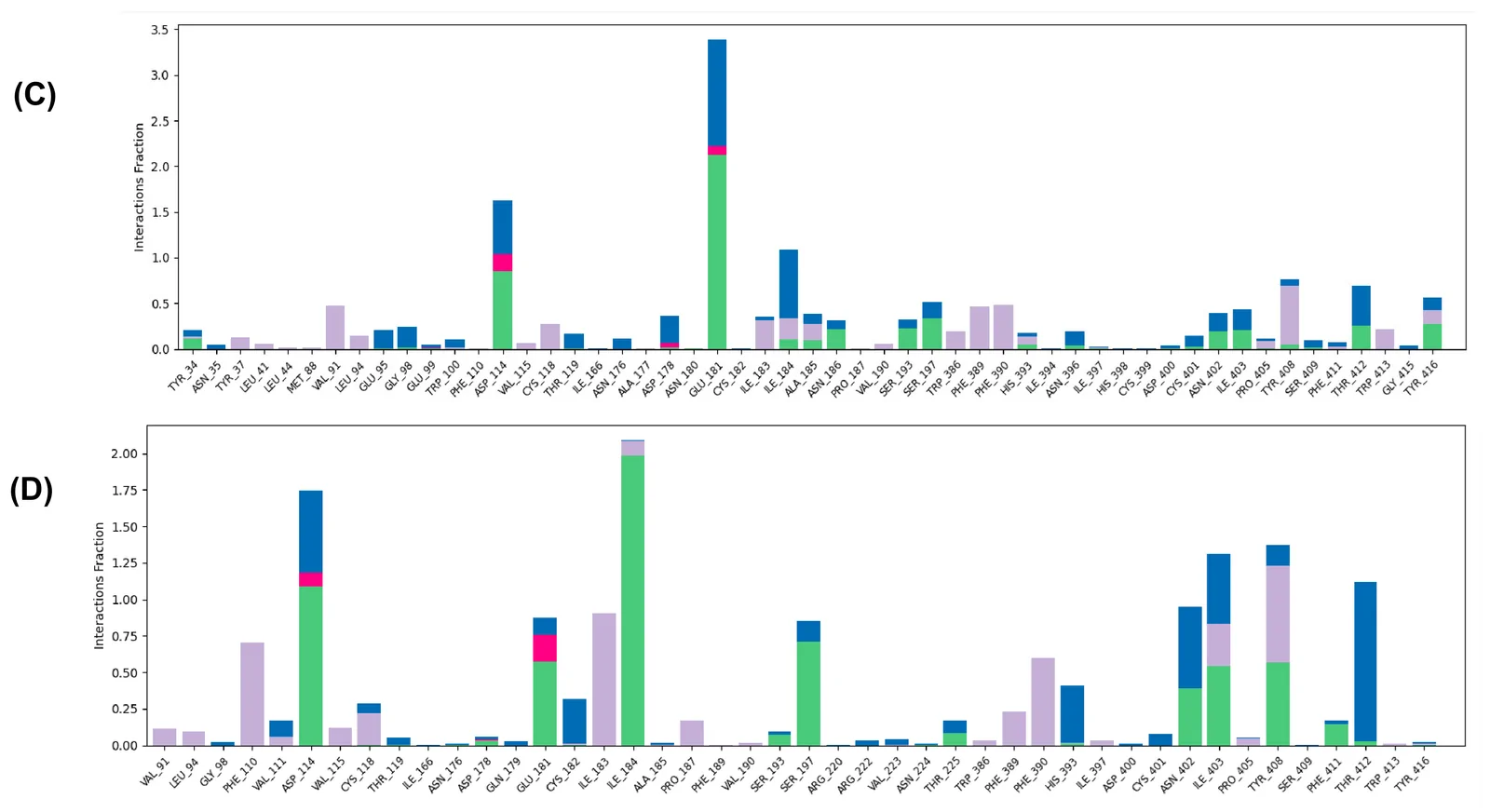
Root-mean-square deviation (RMSD) plots and protein–ligand interaction profiles obtained from 100 ns molecular dynamics simulations of the investigated peptide analogues in complex with the dopamine D2 receptor (D2R). Protein Cα RMSD values are shown in blue, while ligand RMSD values are shown in magenta. Protein–ligand contact histograms present the fraction of simulation time during which individual receptor residues interacted with the ligand. Interactions are classified as hydrogen bonds (green), hydrophobic contacts (purple), ionic interactions (red), and water bridges (blue). (**A**) RMSD analysis for the D2R–KAZO_5.2 complex; (**B**) RMSD analysis for the D2R–KAZO_7.2 complex; (**C**) protein–ligand contacts for the D2R–KAZO_5.2 complex; and (**D**) protein–ligand contacts for the D2R–KAZO_7.2 complex.

To examine the dynamic behavior of the docked complexes, single 100 ns molecular dynamics trajectories were analyzed for KAZO_5.2 and KAZO_7.2 in complex with MOR and D2R (Figure 10 and Figure 11). In each analyzed trajectory, the peptide remained associated with its respective receptor throughout the simulated period. Because independent replicate simulations were not performed, the observations described below are trajectory-specific and should not be interpreted as evidence of statistical reproducibility or convergence. Nevertheless, differences in conformational behavior and receptor–ligand contact patterns were observed among the four analyzed trajectories.

For KAZO_5.2, the MOR trajectory showed comparatively limited receptor and ligand RMSD fluctuations, with receptor RMSD remaining mostly within 2.0–2.6 Å and ligand RMSD fluctuating around 2.5–3.0 Å (Figure 10). Persistent interactions involving ARG211, GLN212, GLU229, ALA304, and GLU310, supported by numerous water-mediated contacts, were maintained during persistent receptor association over the analyzed trajectory. In comparison, the D2R–KAZO_5.2 trajectory showed greater ligand conformational mobility (Figure 11). The receptor RMSD remained mainly within 2.4–3.3 Å, indicating preservation of the overall receptor architecture, whereas the ligand RMSD increased substantially after approximately 35 ns, suggesting conformational rearrangements within the binding pocket. Despite this increased mobility, key interactions involving ASP114 and GLU181 persisted throughout a substantial portion of the trajectory. Additional stabilization was provided by contacts with ILE184, ALA185, ASN402, PRO405, and TYR416, as well as numerous water-mediated interactions. These observations indicate that the increased ligand RMSD reflected reorientation within the receptor cavity rather than dissociation from the binding site.

More pronounced receptor-dependent differences were observed for KAZO_7.2. In the MOR complex (Figure 10), receptor RMSD fluctuated mainly within approximately 2.4–3.3 Å, while the ligand displayed larger conformational rearrangements during the simulation and stabilized at higher RMSD values during the later stages of the trajectory. Contact analysis revealed that the complex was primarily stabilized by interactions involving THR307, LYS303, ALA304, and GLU310, together with hydrophobic contacts and water bridges. In the D2R–KAZO_7.2 trajectory, ligand RMSD values remained largely confined to approximately 1.5–2.5 Å throughout the simulation. Persistent contacts involving ASP114, ILE184, ASN402, ILE403, TYR408, and THR412 accompanied continued receptor association throughout the trajectory. Additional stabilization was provided by extensive hydrophobic contacts with the aromatic region formed by PHE389, PHE390, and TYR408.

Comparison of the four analyzed trajectories suggested receptor-dependent differences in dynamic behavior. In the MOR model, KAZO_5.2 displayed lower conformational variability than KAZO_7.2 during the analyzed 100 ns trajectory, despite the slightly more favorable docking score obtained for KAZO_7.2. In the D2R model, both peptides remained receptor-associated throughout their respective trajectories, with KAZO_7.2 displaying comparatively limited ligand RMSD fluctuations despite receptor conformational rearrangements. These trajectory-specific observations are consistent with, but do not independently validate, the docking-derived hypothesis that MOR may be more sensitive to ligand geometry and conformational organization, whereas D2R may more readily accommodate elongated peptide structures. Across the analyzed D2R trajectories, ASP114, ILE184, PHE389, PHE390, TYR408, and TYR416 were among the residues frequently involved in ligand contacts. Confirmation of these receptor-dependent dynamic patterns will require independent replicate simulations and/or extended conformational sampling.

## 3. Materials and Methods

### 3.1. Cell Lines

Human cancer cell lines A-172 (glioblastoma), A549 (lung adenocarcinoma), SH-SY5Y (neuroblastoma), MDA-MB-231 (triple-negative breast cancer), and HCT116 (colorectal carcinoma), as well as the non-tumorigenic mammary epithelial control line MCF 10A, were used in the study. All cell lines were obtained from the American Type Culture Collection (ATCC; Rockville, MD, USA).

### 3.2. Synthesis and HPLC Analysis of BCM-5 and BCM-7 Hybrid Peptides

Peptides were synthesized by solid-phase peptide synthesis (SPPS) using standard Fmoc/tBu chemistry on Rink amide resin [37]. The synthesis involved iterative cycles of Fmoc deprotection and amino acid coupling. Couplings were performed using equimolar amounts of Fmoc-AA-OH, *N*,*N*-diisopropylcarbodiimide (DIC), and Oxyma Pure, applied in a fivefold excess relative to the resin loading. The reactions were carried out with continuous shaking (Kamush LP360AMP shaker, Zblewo, Poland) for 60 min.

After completion of chain elongation, the peptides were cleaved from the resin and simultaneously deprotected using a cleavage cocktail consisting of trifluoroacetic acid (TFA), triisopropylsilane (TIS), phenol, deionized water, 1,3-dimethoxybenzene, and cis-2,3-dihydroxy-1,4-butanedithiol. The crude peptides were precipitated with cold diethyl ether, dissolved in distilled water, and lyophilized.

Purification was performed by preparative reverse-phase high-performance liquid chromatography (RP-HPLC) using a Phenomenex Gemini-NX C18 column (21.2 × 100 mm, 5 µm, 110 Å). Water and acetonitrile, both containing 0.1% (*v*/*v*) TFA, were used as mobile phases. A linear gradient of 2–70% acetonitrile over 40 min was applied at a flow rate of 20 mL/min. Fractions were collected, analyzed, and combined to obtain high-purity products (>95%).

Purity was assessed by analytical RP-HPLC using a Varian system with a linear gradient of 10–100% acetonitrile in water over 10 min, with both eluents containing 0.1% (*v*/*v*) TFA. The identity of the peptides was confirmed by LC–MS using a Halo C18 column (4.6 × 100 mm, 2.7 µm, 90 Å) using a 1–100% acetonitrile gradient in water over 10 min and a flow rate of 0.5 mL/min (Figure S4). Both mobile phases contained 0.1% (*v*/*v*) formic acid.

### 3.3. Molecular Docking of Casomorphin-Based Compounds to MOR Opioid and D2R Dopamine Receptors

Six peptide ligands were investigated in this study: KAZO_5.1 (Tyr-Pro-Phe-Pro-Gly-Gly-Pro-His-Phe-Met-NH_2_), KAZO_5.2 (Dmt-D-Phe-Phe-Pro-Gly-Gly-His-Phe-Met-NH_2_), KAZO_5.3 (Dmt-Nal(2′)-Phe-Pro-Gly-Gly-His-Phe-Met-NH_2_), KAZO_7.1 (Tyr-Pro-Phe-Pro-Gly-Pro-Ile-Gly-Pro-His-Phe-Met-NH_2_), KAZO_7.2 (Dmt-D-Phe-Phe-Pro-Gly-Pro-Ile-Gly-His-Phe-Met-NH_2_), KAZO_7.3 (Dmt-Nal(2′)-Phe-Pro-Gly-Pro-Ile-Gly-His-Phe-Met-NH_2_). All peptides were constructed using the Peptide Builder module implemented in the Schrödinger Maestro (version 2023-3). The C-termini of all peptides were amidated. Non-standard amino acids, including 2′,6′-dimethyl-L-tyrosine (Dmt), D-phenylalanine (D-Phe), and 2′-naphthylalanine (Nal(2′)), were manually incorporated and geometrically optimized. Protonation states were assigned at physiological pH (7.4 ± 0.5), and ligand structures were further refined using the LigPrep module with the OPLS 2005 force field.

#### 3.3.1. Protein Preparation

The experimental structures of the murine μ-opioid receptor (MOR) and human dopamine D2 receptor (D2R) were retrieved from the Protein Data Bank (PDB). The MOR structure (PDB ID: 5C1M) corresponds to the active *Mus musculus* receptor bound to the agonist BU72, whereas the D2R structure (PDB ID: 7JVR) represents the human D2R-containing receptor complex bound to bromocriptine and G_i_ protein. Murine and human MOR are highly conserved, with approximately 94% sequence identity across the complete receptor sequence and approximately 99% identity within the structurally resolved receptor region. Accordingly, 5C1M was retained as a structural surrogate for the MOR docking analysis; however, all residue-level MOR descriptions in the present work refer specifically to the murine 5C1M structure. Protein structures were prepared using the Protein Preparation Wizard in Schrödinger Maestro. All crystallographic water molecules, co-crystallized ligands (after grid generation), ions, and non-receptor components were removed. In the case of D2R, G protein subunits and stabilizing antibody fragments were deleted, retaining only the receptor chain. Missing hydrogen atoms were added, bond orders were assigned, disulfide bonds were created, and protonation states were adjusted at pH 7.0. The structures were subsequently optimized and energy-minimized using the OPLS 2005 force field until convergence.

#### 3.3.2. Receptor Grid Generation

Receptor grids were generated using the Glide module. The grid centers were defined based on the coordinates of the co-crystallized ligands (BU72 for MOR and bromocriptine for D2R). A cubic grid box was generated with dimensions sufficient to accommodate peptide ligands, and the “dock ligand with length” parameter was set to 20 Å. Default van der Waals scaling (1.0) and partial charge cutoff (0.25) values were applied.

#### 3.3.3. Molecular Docking

Flexible protein–peptide docking was performed using the Glide SP-Peptide docking protocol implemented in Schrödinger Maestro [38,39]. This protocol enables enhanced conformational sampling suitable for large and flexible ligands such as peptides. For each ligand–receptor pair, multiple docking poses were generated, and the top-ranking conformations were selected based on GlideScore values and visual inspection of binding modes. Attention was paid to the orientation of N-terminal pharmacophoric residues and the formation of key interactions within the receptor binding pocket.

#### 3.3.4. Docking Protocol Validation

To assess the ability of the docking setup to reproduce experimentally observed binding modes, the co-crystallized ligands were redocked into their corresponding prepared receptor structures using the receptor grids described above. BU72 was redocked into MOR (PDB ID: 5C1M), whereas bromocriptine was redocked into D2R (PDB ID: 7JVR). The resulting poses were superimposed onto the corresponding crystallographic ligand poses, and heavy-atom RMSD values were calculated between the redocked and crystallographic conformations. Heavy-atom RMSD values of 0.410 Å for BU72 and 0.915 Å for bromocriptine were obtained.

### 3.4. Evaluation of Cell Viability with the MTT Assay

#### 3.4.1. Culture Conditions

HCT116 and A172 cells were cultured in Dulbecco’s Modified Eagle’s Medium (DMEM) supplemented with L-glutamine. MDA-MB-231 cells were maintained in Iscove’s Modified Dulbecco’s Medium (IMDM) supplemented with L-glutamine and 25 mM HEPES. A549 cells were cultured in F-12K (Kaighn’s modification of Ham’s F-12) medium (Gibco, Thermo Fisher Scientific, Waltham, MA, USA). SH-SY5Y cells were maintained in ready-to-use DMEM/F-12 medium (Biowest, Nuaillé, France). Unless otherwise specified, culture media were supplemented with 10% heat-inactivated fetal bovine serum (FBS; Biowest, Nuaillé, France), 1% (*v*/*v*) MEM non-essential amino acids (NEAA; Biowest, Nuaillé, France), and 1% (*v*/*v*) antibiotic–antimycotic solution (Biowest, Nuaillé, France). MCF 10A cells were cultured in F-12K (Kaighn’s modification of Ham’s F-12) medium (Gibco, Thermo Fisher Scientific, Waltham, MA, USA) supplemented with 5% heat-inactivated FBS (Biowest, Nuaillé, France), 1% (*v*/*v*) MEM non-essential amino acids, 1% (*v*/*v*) antibiotic–antimycotic solution, 5 μg/mL insulin, 0.04 μg/mL hydrocortisone (Merck, Darmstadt, Germany), and 15 ng/mL human epidermal growth factor (hEGF; PeproTech, Cranbury, NJ, USA). All cells were maintained at 37 °C in a humidified atmosphere containing 5% CO_2_ and were routinely cultured in the exponential growth phase.

#### 3.4.2. MTT Assay

Cell viability of treated cells was determined using the MTT assay [40], based on the reduction in tetrazolium salt by mitochondrial dehydrogenases. The tested compounds (KAZO_5-series and KAZO_7-series) were dissolved in dimethyl sulfoxide (DMSO) to prepare stock solutions and subsequently diluted in culture medium to obtain the desired working concentrations.

Cells were seeded in 96-well plates at densities allowing sub-confluent growth after 24 and 48 h. Following attachment, cells were treated with the tested compounds (10–300 µg/mL) for 24 h and 48 h. After incubation, MTT solution was added to each well, and cells were incubated under standard conditions to allow formazan formation. The medium was then removed, the formazan crystals were dissolved in isopropanol, and absorbance was measured at 570 nm using a PowerWave XS microplate reader (BioTek, Winooski, VT, USA).

Each experimental condition (cell line, compound, concentration, and incubation time) was evaluated in three independent biological experiments, each performed in quadruplicate. Thus, each viability value represents the mean of 12 measurements. Vehicle-treated controls were included separately for each cell line, incubation time, and biological replicate to minimize inter-experimental variability.

For the most active compounds, the contribution of the MOR-related pharmacophore to the hybrid-induced antiproliferative activity was assessed. In this context, cells were pre-incubated with 10 µg/mL naloxone (NAL; Sigma-Aldrich, Poznań, Poland) for 1 h under standard culture conditions. Following pre-treatment, the medium was replaced with fresh medium containing NAL (10 µg/mL) together with the tested compounds at the indicated concentrations (200 µg/mL). After 48 h of incubation, cell viability was assessed using the MTT assay as described above. Vehicle-treated control groups were included to account for solvent effects.

### 3.5. Analysis of Cell Cycle Distribution and Apoptosis

Apoptosis was evaluated using Annexin V-FITC/Propidium Iodide (PI) staining according to the manufacturer’s instructions. Cells were seeded in 8-well glass-bottom chamber slides (Ibidi, Bavaria, Germany) and exposed to selected concentrations of the tested compounds, as indicated in the Results section. Following incubation, cells were stained with Annexin V-FITC and Propidium Iodide for 15 min at room temperature in the dark. Fluorescence images were acquired immediately using a fluorescence microscope (FluoView 500/IX70, Olympus Corporation, Tokyo, Japan) equipped with appropriate FITC and PI filter sets. Images were captured from randomly selected fields using identical exposure settings for all experimental groups.

Cell cycle distribution was assessed by flow cytometry following DNA staining with propidium iodide (PI). Human colorectal cancer HCT116 cells were treated with compounds 5.2 and 7.2 (200 µg/mL) for the indicated time. Cells were harvested, washed with cold PBS, and fixed in 70% ice-cold ethanol at −20 °C for at least 24 h. After fixation, cells were incubated with PI (50 µg/mL) and RNase A (100 µg/mL) for 30 min at room temperature in the dark. Data acquisition was performed using a flow cytometer, collecting at least 10,000 events per sample. Doublets and debris were excluded based on FSC/SSC and pulse-width gating. Cell cycle distribution (G0/G1, S, G2/M) was determined from DNA content histograms using MultiCycle AV for Windows (version 300) software. Results were expressed as the percentage of cells in each phase.

### 3.6. Assessment of Hemolytic Activity of Hybrid Peptides

The compounds’ hemolytic activity was measured using a modified Laskowska et al. protocol [22]. Defibrinated sheep blood was obtained from BioMaxima (Lublin, Poland). Whole blood was centrifuged at 2000 rpm for 10 min at 4 °C. The supernatant was carefully aspirated without disturbing the red blood cell (RBC) pellet. The RBCs were resuspended in PBS and centrifuged again at 2000 rpm for 10 min. This washing step was repeated three times. After the final wash, the supernatant was removed, and the RBC pellet was diluted 1:50 in PBS (pH 7.4, room temperature) to obtain a 2% RBC suspension. The positive control consisted of sterile distilled water, while the negative control was PBS (pH 7.4). The 2% RBC suspension was then incubated with serial dilutions of the compounds (50–500 µg/mL) at a 1:1 ratio for 1 h at 37 °C. Following incubation, samples were centrifuged at 4000 rpm for 5 min at 4 °C, and 100 µL of the supernatant from each sample was transferred to a 96-well plate. Optical density (OD) was measured at 540 nm using a microplate reader.

Hemolysis induced by the compound was calculated using the following formula: Hemolysis (%) = [(A − A_0_%)/(A_100_% − A_0_%)] × 100%, where A—absorbance of the sample incubated with the compound; A_100_%—absorbance of the positive control; A_0_%—absorbance of the negative control.

### 3.7. Enzymatic Stability Assay

#### 3.7.1. LC-MS Conditions

The LC-MS/MS was performed using a Dionex Ultimate 3000RS device (Dionex, Sunnyvale, CA, USA) equipped with an autosampler, column oven, and degasser. The chromatograph was coupled with a Bruker Amazon SL ion trap mass spectrometer (Bruker Daltonik, Bremen, Germany) without splitting.

The separation was carried out using a Kinetex XB-C_18_ column (150 mm × 2.1 mm × 1.7 μm; Phenomenex, Torrance, CA, USA). The mobile phase consisted of A: 0.1% formic acid in deionized water and B: 0.1% formic acid in acetonitrile (MeCN). The two-step gradient was used from 5% B to 50% B in 13 min and from 50% B to 65% B up to 20 min. The column temperature was maintained at 25 °C with a flow rate equal to 0.3 mL/min. The eluate was introduced directly to the mass spectrometer. The ion trap setting was as follows: capillary voltage 4500 V, endplate offset 500 V, nebulizer pressure 40 psi, drying gas temperature 145 °C, and gas flow rate 9 L/min. The instrument used a smart parameter setting (SPS) fixed at 1000 amu. The scan range was from *m*/*z* = 70 to *m*/*z* = 2200. Compounds were analyzed in negative ion mode.

#### 3.7.2. In Vitro Stability in Human Plasma

Human plasma samples were used in this study. Stock solutions of the tested compounds (10 mg/mL) were diluted ten-fold with ultrapure water by mixing 20 µL of the stock solution with 180 µL of water to obtain working solutions at a concentration of 1 mg/mL. Aliquots of 450 µL of human plasma were transferred into 1.5 mL microcentrifuge tubes and preincubated at 37 °C using a thermoblock. Subsequently, 50 µL of the compound working solution (1 mg/mL) was added to each tube, resulting in a final incubation volume of 500 µL. The samples were mixed immediately after compound addition.

To determine the initial concentration (time 0), a 25 µL aliquot was withdrawn immediately after mixing and transferred into a microcentrifuge tube containing 175 µL of acetonitrile supplemented with 0.1% formic acid to terminate the reaction and precipitate proteins. The mixture was vortex-mixed and stored at 4 °C for at least 15 min before further processing. Additional 25 µL aliquots were collected at predetermined time points of 1, 2, 3, 4, 5, 7, 10, 15, 20, 30, 45, and 60 min, as well as 2, 4, 7, and 24 h. Each aliquot was immediately quenched with 175 µL of acetonitrile containing 0.1% formic acid and mixed thoroughly.

Following protein precipitation, all samples were centrifuged at 15,000× *g* for 15 min. Subsequently, 50 µL of the clear supernatant was transferred into HPLC autosampler vials equipped with glass inserts for chromatographic analysis. An injection volume of 4 µL was used for each analysis.

### 3.8. Stability in Cell Culture Medium

This analysis was prompted by morphological abnormalities observed under microscopic inspection of KAZO_7.3-treated HCT116 cells during the MTT assay (Figure S2C), raising concern that the compound’s pronounced viability-reducing effect might be attributable to degradation products rather than the intact peptide. The stability of the KAZO_7.3 peptide was evaluated in Dulbecco’s Modified Eagle Medium/Nutrient Mixture F-12 (DMEM/F12) supplemented with 10% fetal bovine serum (FBS) and 1% penicillin–streptomycin. A stock solution of the peptide was first prepared in dimethyl sulfoxide (DMSO) and subsequently diluted into the culture medium to obtain a final peptide concentration of 300 µg/mL.

The peptide-containing medium was incubated at 37 °C in a humidified incubator with 5% CO_2_. Aliquots (25 µL) were collected immediately after preparation (0 h) and after 8 h and 24 h of incubation. Each sample was immediately mixed with 175 µL of acetonitrile to precipitate proteins. The mixture was vortex-mixed and stored at 4 °C for at least 15 min before further processing. Following protein precipitation, all samples were centrifuged at 15,000× *g* for 15 min. Subsequently, 50 µL of the clear supernatant was transferred into HPLC autosampler vials equipped with glass inserts for chromatographic analysis. An injection volume of 3 µL was used for each analysis.

### 3.9. Western Blot Analysis

The level of opioid receptor Mu 1 (OPRM1) protein in the HCT116 cell line after KAZO_5.2 and KAZO_7.2 treatment was evaluated by Western blot according to the manufacturer’s protocol. Cells were seeded in 6-well plates and, after 24 h, treated with the tested compounds at 200 µg/mL in duplicates; control cells received vehicle (DMSO) for 24 h. Cells were lysed in RIPA buffer supplemented with protease and phosphatase inhibitor cocktail (Thermo Fisher Scientific, Waltham, MA, USA). Whole-cell lysates were clarified by centrifugation at 16,000× *g*, 4 °C, 30 min, and protein concentration was determined using the BCA Protein Assay Kit (Pierce, Thermo Fisher Scientific, Waltham, MA, USA) according to the manufacturer’s protocol.

Equal amounts of protein were resolved by SDS-PAGE using a vertical electrophoresis unit Mini-PROTEAN^®^ 3 Cell (Bio-Rad, Hercules, CA, USA) and transferred onto nitrocellulose membranes with the Trans-Blot^®^ Turbo system (Bio-Rad, Hercules, CA, USA). Membranes were blocked (e.g., 5% BSA in TBST, 1 h, RT) and incubated with primary monoclonal antibodies: anti-OPRM1 (Affinity Biosciences, Cincinnati, OH, USA) and anti-GAPDH (Thermo Fisher Scientific, Waltham, MA, USA). GAPDH served as the loading control. After washing, membranes were incubated with the appropriate fluorescent detection reagents from the WesternDot™ kit (Thermo Fisher Scientific, Waltham, MA, USA) following the manufacturer’s instructions. Signals were captured using a Gel Pro 230 (DNR Cleaver Scientific, Warwickshire, UK).

### 3.10. In Silico Molecular Dynamics Analysis of Selected Casomorphin-Based Hybrid Peptides

The best-scoring docking poses of selected compounds (i.e., KAZO_5.2 and KAZO_7.2; based on molecular docking) were subjected to molecular dynamics (MD) simulations using the Desmond module of Schrödinger Maestro [41]. These analogues were selected because they exhibited the most favorable docking scores toward MOR and D2R among the investigated series. Each receptor–ligand complex was embedded in a pre-equilibrated DPPC membrane and solvated using the TIP3P water model in an orthorhombic box with a 10 Å buffer. Systems were neutralized by adding appropriate counterions (Cl^−^). The systems were relaxed using a standard multi-stage equilibration protocol, including energy minimization, gradual heating to 300 K, and pressure equilibration. Production MD simulations were performed under the NPT ensemble at 300 K and 1 atm for 100 ns using the Nose–Hoover thermostat and Martyna–Tobias–Klein barostat. A 2.0 fs time step was used, and trajectories were recorded at 100 ps intervals. One 100 ns production trajectory was generated for each receptor–ligand complex. Independent replicate simulations were not performed; accordingly, the MD analysis was treated as descriptive and trajectory-specific.

### 3.11. Data Analysis

Experimental data were analyzed and graphically processed using the GraphPad Prism software version 9.0 (GraphPad Prism Software Inc., San Diego, CA, USA).

As IC50 values could not be determined within the tested concentration range for either cell type, a classical selectivity index (SI = IC50normal/IC50cancer) could not be calculated. Therefore, as a novel study-specific complementary measure of concentration- and time-matched selectivity, a selectivity ratio (SR) was calculated as SR = (% viability of HCT116 cells)/(% viability of MCF 10A cells) at 200 and 300 µg/mL after 24 h and 48 h.

All data are presented as mean ± SD. Statistical analyses were performed using GraphPad Prism (version 9.0; GraphPad Prism Software Inc., San Diego, CA, USA). The following tests were applied: one-way ANOVA followed by Tukey’s multiple comparisons test, and two-way ANOVA followed by Sidak’s or Bonferroni’s multiple comparisons test. Differences were considered statistically significant at *p* < 0.05.

## 4. Conclusions 

The present study constitutes a preliminary investigation into the potential of casomorphin–ranatensin hybrid peptides as dual-target agents that may affect cancer cells. While the obtained results are encouraging, they should be interpreted with caution given the early-stage nature of the work.

Molecular docking and molecular dynamics simulations provided a computational rationale for the dual-target design and supported the structural plausibility of interactions with both MOR and D2R binding sites. These calculations, however, do not constitute experimental evidence of receptor binding or functional receptor engagement. Among the evaluated analogues, KAZO_5.2 and KAZO_7.2 displayed selective reduction in HCT116 colorectal cancer cell viability with negligible effects on non-tumorigenic cells and no significant hemolytic activity. The partial attenuation of these effects by naloxone is consistent with, although not conclusive evidence for, the involvement of opioid-sensitive pathways. Both compounds demonstrated high proteolytic stability in human plasma, which is a necessary but not sufficient condition for further pharmacological development.

Taken together, the present findings suggest that the casomorphin–ranatensin hybrid approach merits further investigation, but substantial additional work is required before any conclusions regarding therapeutic potential can be drawn.

In this context, several limitations of the present study should be acknowledged. First, we did not determine receptor binding affinity at MOR and D2R, as it was not within the scope of the present work, and the mechanistic basis of the observed antiproliferative effects remains to be established; radioligand binding assays, selective receptor antagonists, siRNA-mediated knockdown, and downstream signaling analyses will be necessary to elucidate the receptor-level mechanism of action. The MD simulations were limited to a single 100 ns trajectory for each receptor–ligand complex and did not include independent replicate runs. Consequently, the present simulations do not establish statistical reproducibility or convergence of the observed dynamic behavior. The receptor-dependent patterns described here should therefore be regarded as trajectory-specific, hypothesis-generating observations rather than general properties of the investigated complexes. Confirmation of these observations will require independent replicate simulations and/or extended conformational sampling. Second, the MTT assay reflects overall cellular metabolic activity rather than cell death per se (MTT reduction is not exclusively mitochondrial), and the absence of pronounced apoptosis or cell cycle arrest suggests cytostatic rather than cytotoxic effects at the tested concentrations; complementary assays are needed for more precise characterization. Third, and importantly, IC50 values could not be determined for either compound within the tested concentration range, as 50% reduction in cell viability was not achieved; biologically relevant effects were observed only at relatively high peptide concentrations, which substantially limits quantitative assessment of antiproliferative potency. Notably, to date no peptide-based compound with a structurally analogous dual-target opioid/dopaminergic design has received clinical approval, which precludes the use of an appropriate reference standard. This, in turn, highlights the need for structural optimization aimed at improving cellular potency before meaningful structure–activity relationships can be established. Fourth, MOR/OPRM1 protein levels were assessed qualitatively by Western blot without densitometric quantification; therefore, potential treatment-induced changes in receptor expression could not be formally evaluated. Additionally, the evaluation was limited to a small panel of cell lines, and no in vivo studies were conducted, which substantially restricts the conclusions that can be drawn regarding the therapeutic relevance of the present findings. Addressing these limitations will be essential before the casomorphin–ranatensin hybrid scaffold can be meaningfully advanced toward preclinical development.

## Figures and Tables

**Table 1:** Glide docking scores calculated for the investigated peptide ligands in complexes with the μ-opioid receptor (MOR) and dopamine D2 receptor (D2R). More negative GlideScore values indicate more favorable predicted docking according to the applied scoring function and were used here for relative ranking within the investigated ligand series. These values should not be interpreted as experimentally determined binding affinities or binding free energies.

Compound	GlideScore MOR (5C1M)	GlideScore D2R (7JVR)
**KAZO_5.1**	−11.635	−15.533
**KAZO_5.2**	−12.989	−15.513
**KAZO_5.3**	−12.904	−15.672
**KAZO_7.1**	−11.497	−14.673
**KAZO_7.2**	−13.184	−15.987
**KAZO_7.3**	−11.815	−15.118

## Data Availability

The data are included in the text.

## Supplementary Materials

The following supporting information can be downloaded at https://www.mdpi.com/article/10.3390/molecules31183221/s1, Figure S1: Validation of the molecular docking setup by redocking of the co-crystallized ligands. (A) Superposition of the crystallographic and redocked poses of BU72 in the μ-opioid receptor (MOR, PDB ID: 5C1M). (B) Superposition of the crystallographic and redocked poses of bromocriptine in the dopamine D2 receptor (D2R, PDB ID: 7JVR). Crystallographic ligand poses are shown in green and redocked poses in red. The corresponding heavy-atom RMSD values were 0.410 Å for BU72 and 0.915 Å for bromocriptine; Figure S2: (A) Representative structures of putative KAZO_7.3 degradation products detected in the culture medium used for the MTT assay, based on the observed molecular masses. (B) LC-MS chromatograms of KAZO_7.3-containing culture medium collected immediately after preparation (0 h) and after 24 h of incubation under cell culture conditions. Major detected ions are indicated by their m/z values. Compounds were identified as Dmt-Nal(2′)-FPGPIGH [m/z observed = 1110.9]; and Dmt-Nal(2′) [m/z observed = 405.2] with KAZO_7.3 [m/z observed = 1388.1]. C) Representative image of an MTT assay plate showing HCT116 cells treated with KAZO_7.3 at a concentration of 300 µg/mL; Figure S3: Western blot analysis of OPRM1 (MOR) protein in HCT116 cancer cells following treatment with KAZO_5.2 and KAZO_7.2 (200 μg/mL); Figure S4: HPLC (left panel) and LC-MS (right panel) chromatograms for synthesized casomorphin-based hybrid peptides: (A) KAZO_5.1; (B) KAZO_5.2; (C) KAZO_5.3; (D) KAZO_7.1; (E) KAZO_7.2; (F) KAZO_7.3.
