# Supplementary material for: Rational Design of Putative Dual-Target Opioid-Dopaminergic Casomorphin–Ranatensin Hybrid Peptides: Computational Modeling and Preliminary Antiproliferative Evaluation
Source: Molecules. 2026 Sep 12;31(18):3221. doi: 10.3390/molecules31183221 (PMC13609293; doi:10.3390/molecules31183221)
Supplement: Supplementary file 1 [file molecules-31-03221-s001.zip › molecules-4509894-supplementary.pdf]

Supplementary

A)

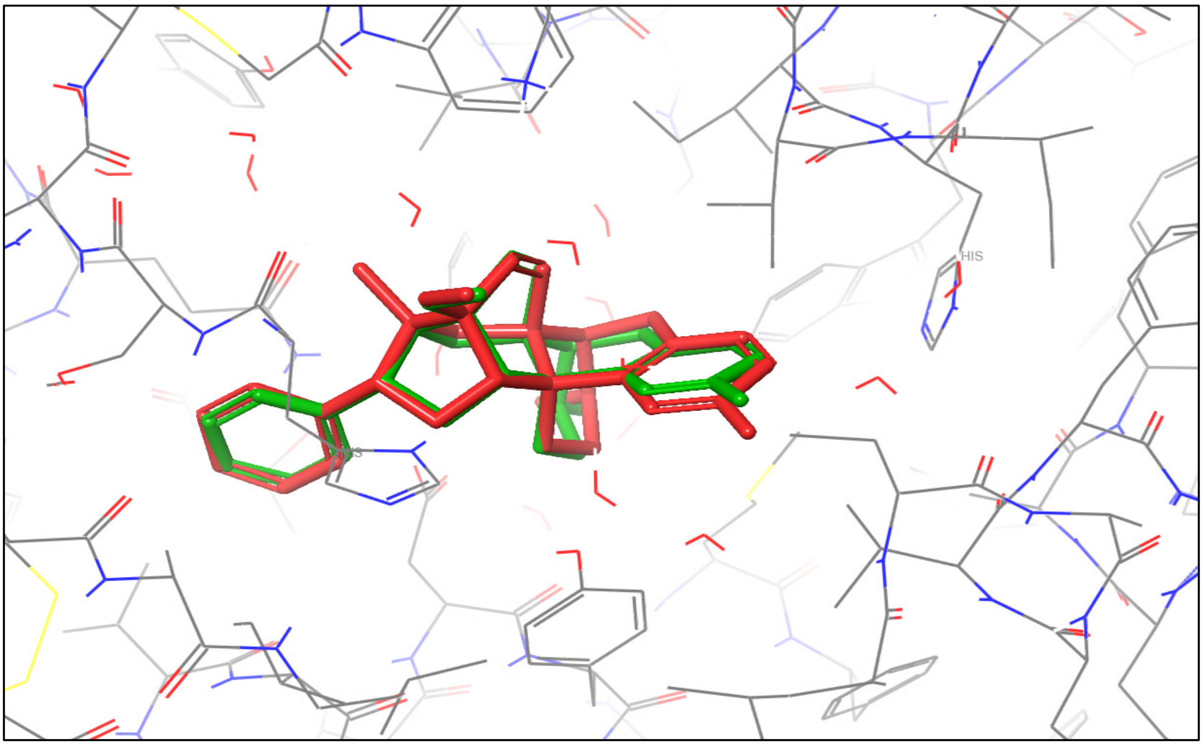

B)

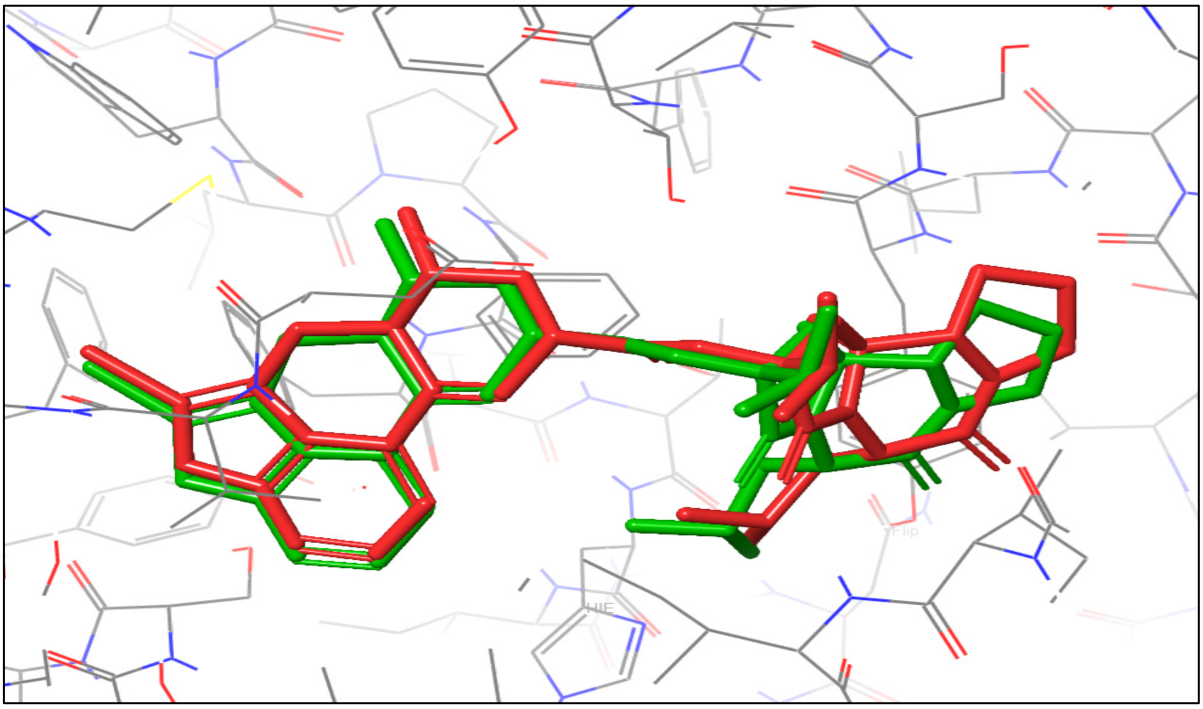

Figure S1. Validation of the molecular docking setup by redocking of the co-crystallized ligands. (A) Superposition of the crystallographic and redocked poses of BU72 in the μ-opioid receptor (MOR, PDB ID: 5C1M). (B) Superposition of the

crystallographic and redocked poses of bromocriptine in the dopamine D2 receptor (D2R, PDB ID: 7JVR). Crystallographic ligand poses are shown in green and redocked poses in red. The corresponding heavy-atom RMSD values were 0.410 Å for BU72 and 0.915 Å for bromocriptine.

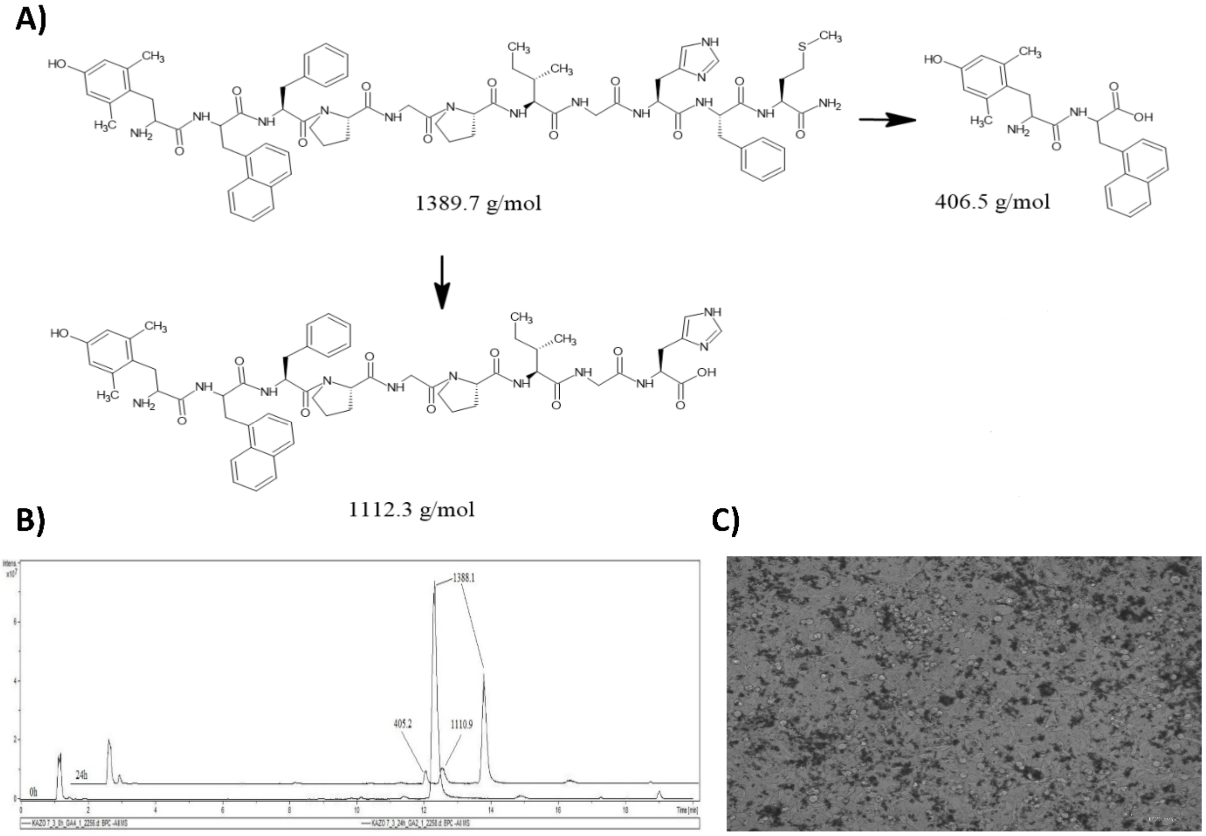

Figure S2. A) Representative structures of putative KAZO_7.3 degradation products detected in the culture medium used for the MTT assay, based on the observed molecular masses. **B)** LC-MS chromatograms of KAZO_7.3-containing culture medium collected immediately after preparation (0 h) and after 24 h of incubation under cell culture conditions. Major detected ions are indicated by their *m*/*z* values. Compounds were identified as Dmt-Nal(2')-FPGPIGH [m/z observed = 1110.9]; and Dmt-Nal(2') [m/z observed = 405.2] with KAZO_7.3 [m/z observed = 1388.1]. **C)** Representative image of an MTT assay plate showing HCT116 cells treated with KAZO_7.3 at a concentration of 300 μg/mL.

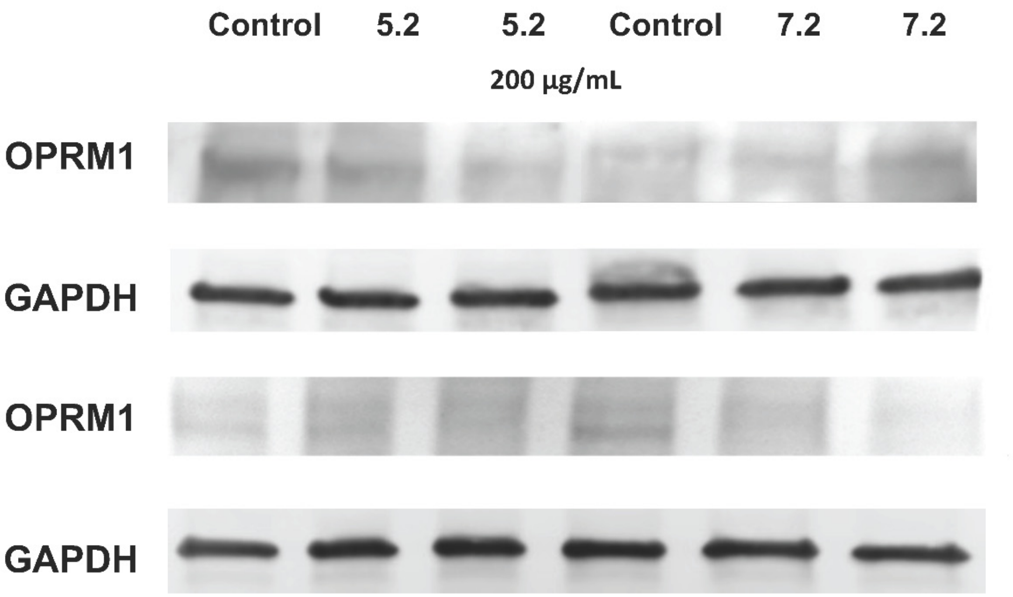

Figure S3. Western blot analysis of OPRM1 (MOR) protein in HCT116 cancer cells following treatment with KAZO_5.2 and KAZO_7.2 (200 μg/mL).

A)

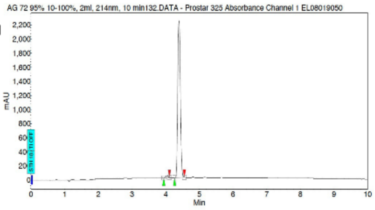

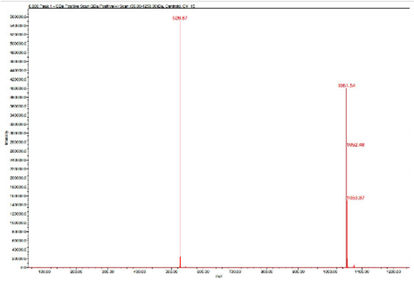

Peak results :

Index	Name	Time [Min]	Quantity [% Area]	Height [mAU]	Area [mAU.Min]	Area % [%]
1	UNKNOWN	4.09	1.10	28.6	2.3	1.098
2	UNKNOWN	4.39	98.90	2224.4	203.8	98.902
Total			100.00	2253.0	206.0	100.000

B)

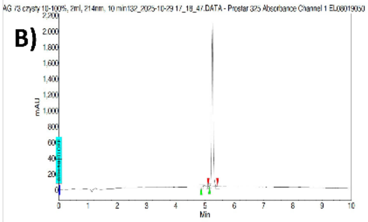

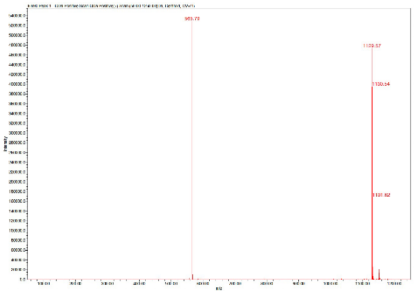

Peak results :

Index	Name	Time [Min]	Quantity [% Area]	Height [mAU]	Area [mAU.Min]	Area % [%]
1	UNKNOWN	4.98	0.76	17.4	0.9	0.755
2	UNKNOWN	5.25	99.24	2063.8	116.3	99.245
Total			100.00	2081.2	117.2	100.000

C)

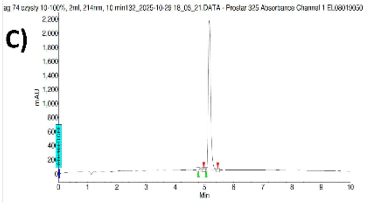

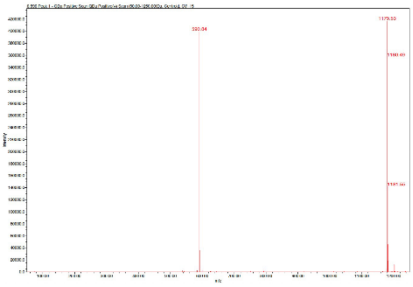

Peak results :

Index	Name	Time [Min]	Quantity [% Area]	Height [mAU]	Area [mAU.Min]	Area % [%]
1	UNKNOWN	4.87	0.68	18.3	1.4	0.675
2	UNKNOWN	5.17	99.32	2125.4	212.4	99.325
Total			100.00	2143.7	213.9	100.000

D)

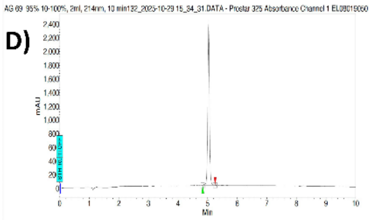

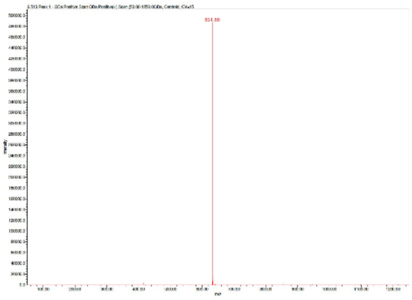

Peak results :

Index	Name	Time [Min]	Quantity [% Area]	Height [mAU]	Area [mAU.Min]	Area % [%]
1	UNKNOWN	5.03	100.00	2386.1	150.8	100.000
Total			100.00	2386.1	150.8	100.000

E)

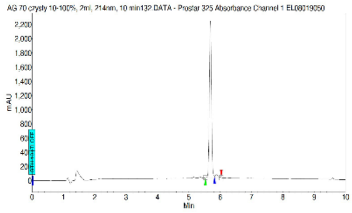

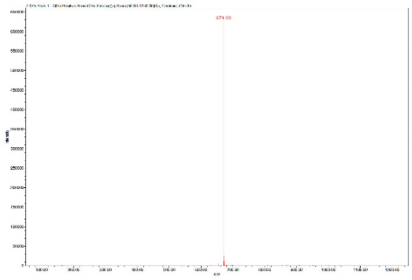

Peak results :

Index	Name	Time [Min]	Quantity [% Area]	Height [mAU]	Area [mAU.Min]	Area % [%]
1	UNKNOWN	5.69	97.36	2203.7	169.9	97.363
2	UNKNOWN	5.86	2.64	48.1	4.6	2.637
Total			100.00	2251.8	174.5	100.000

F)

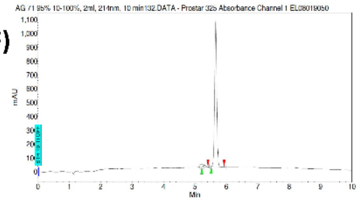

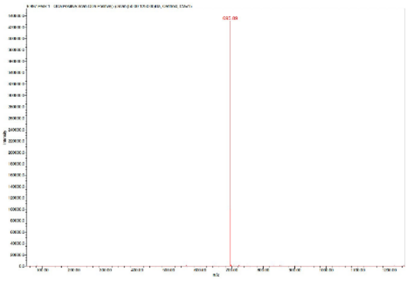

Peak results :

Index	Name	Time [Min]	Quantity [% Area]	Height [mAU]	Area [mAU.Min]	Area % [%]
1	UNKNOWN	5.31	1.20	11.6	1.0	1.196
2	UNKNOWN	5.66	98.80	1057.6	82.3	98.804
Total			100.00	1069.2	83.3	100.000

Figure S4. HPLC (left panel) and LC-MS (right panel) chromatograms for synthesized casomorphin-based hybrid peptides: A) KAZO_5.1; B) KAZO_5.2; C) KAZO_5.3; D) KAZO_7.1; E) KAZO_7.2; F) KAZO_7.3
